# A glance at subgenomic flavivirus RNAs and microRNAs in flavivirus infections

**DOI:** 10.1186/s12985-016-0541-3

**Published:** 2016-05-28

**Authors:** Lorena Bavia, Ana Luiza Pamplona Mosimann, Mateus Nóbrega Aoki, Claudia Nunes Duarte dos Santos

**Affiliations:** Laboratório de Virologia Molecular, Instituto Carlos Chagas (ICC/FIOCRUZ-PR), Rua Prof. Algacyr Munhoz Mader 3775, CIC, CEP: 81350-010 Curitiba, Paraná Brazil

**Keywords:** Flavivirus, MicroRNAs, Subgenomic flavivirus RNAs, Untranslated region, Viral replication

## Abstract

The family *Flaviviridae* comprises a wide variety of viruses that are distributed worldwide, some of which are associated with high rates of morbidity and mortality. There are neither vaccines nor antivirals for most flavivirus infections, reinforcing the importance of research on different aspects of the viral life cycle. During infection, cytoplasmic accumulation of RNA fragments mainly originating from the 3′ UTRs, which have been designated subgenomic flavivirus RNAs (sfRNAs), has been detected. It has been shown that eukaryotic exoribonucleases are involved in viral sfRNA production. Additionally, viral and human small RNAs (sRNAs) have also been found in flavivirus-infected cells, especially microRNAs (miRNAs). miRNAs were first described in eukaryotic cells and in a mature and functional state present as single-stranded 18–24 nt RNA fragments. Their main function is the repression of translation through base pairing with cellular mRNAs, besides other functions, such as mRNA degradation. Canonical miRNA biogenesis involves Drosha and Dicer, however miRNA can also be generated by alternative pathways. In the case of flaviviruses, alternative pathways have been suggested. Both sfRNAs and miRNAs are involved in viral infection and host cell response modulation, representing interesting targets of antiviral strategies. In this review, we focus on the generation and function of viral sfRNAs, sRNAs and miRNAs in West Nile, dengue, Japanese encephalitis, Murray Valley encephalitis and yellow fever infections, as well as their roles in viral replication, translation and cell immune response evasion. We also give an overview regarding other flaviviruses and the generation of cellular miRNAs during infection.

## Background

MicroRNAs (miRNAs) are a class of molecules first observed in 1993 in *Caenorhabditis elegans*, where it was shown that the level of LIN-14 protein was regulated by a 22 nucleotide (nt) small RNA (sRNA) transcript generated from the gene *lin-4* [1]. Further studies in *Caenorhabditis elegans* characterized a second sRNA originating from the gene *let-7*, which suppressed *lin-14*, *lin-28*, *lin-41*, *lin-42* and *daf-12* heterochronic gene expression [2]. Moreover, it was observed that the let-7 sRNA sequence is conserved among many species, suggesting a putative function for this sRNA [3]. These small single-stranded RNA molecules (18–21 nt) that were first described as sRNA are presently known as miRNAs [4].

Eukaryotic miRNAs are generated by transcription of the cellular genome through different pathways, and more than 2000 human miRNAs have already been identified [5]. In addition, there is evidence for 1098 novel human miRNA candidates [6]. miRNA genes (miR genes) are located in variable genome regions, such as the intronic or exonic sequences of protein-coding genes and intronic or exonic sequences of noncoding RNAs [7]. The majority of intronic miRNAs within protein coding genes are transcribed by the same promoter of the gene. However, approximately one-third of intronic miRNA transcripts have independent promoters, indicating that they may be transcribed by a separate and controlled mechanism or pathway [8]. In this regard, common features of known RNA polymerase II and III promoters, such as transcription start sites, CpG islands, conserved transcription factor binding sites and A/B box sequences, have been identified upstream of intronic miRNAs and are predicted to function independent of host gene transcription [9]. All these features indicate that miRNA expression may be regulated by transcription factors, enhancers, silencing elements and chromatin modification [10].

miRNAs can be found in plants, fungi, mammals and viruses [11]. In viruses, miRNA production depends on the eukaryotic cell machinery and viral genome composition: DNA or RNA [12]. The first report that described miRNAs of viral origin used a cell line infected with Epstein-Barr virus, a DNA virus [13]. Since this report, studies employing DNA viruses to investigate viral miRNA generation and function have increased substantially. On the other hand, there are fewer studies investigating viral miRNA from cells infected with RNA viruses [14–16]. Therefore, we gathered here the published information on non-coding RNA (viral subgenomic RNAs, viral small RNAs, viral miRNAs and human miRNAs) synthesis and function during flavivirus infections. First, it is important to review some concepts on miRNA biosynthesis and function in eukaryotic cells.

## Eukaryotic microRNA biogenesis and functions

### miRNA biogenesis

**Fig. 1:**
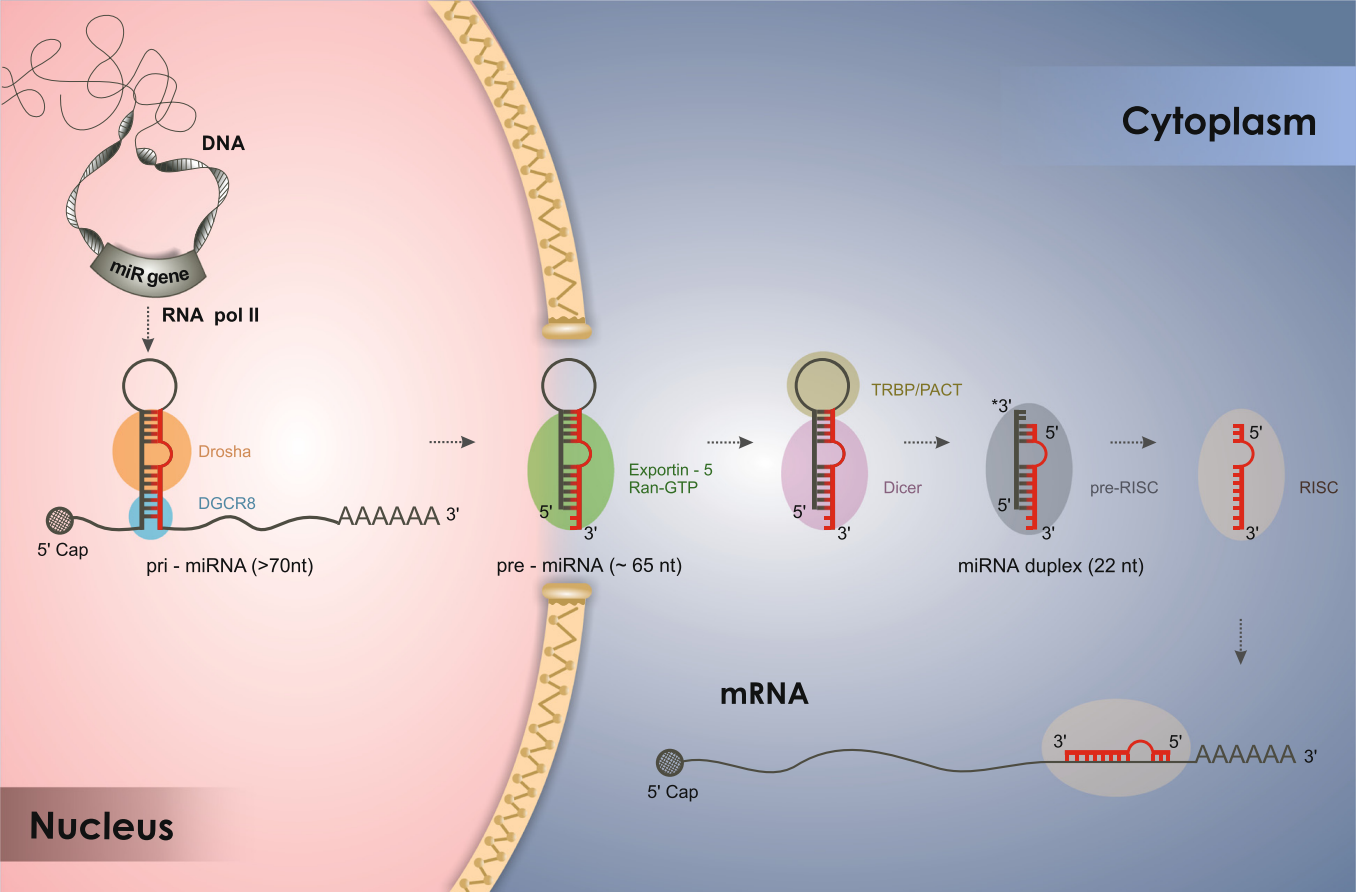
Canonical miRNA biogenesis. miRNAs are transcribed by RNA polymerase II from miR genes into a pri-miRNA transcript (> 70 nt), which is metabolized by a microprocessor complex (Drosha and DGCR8), forming the pre-miRNA (~65 nt). The pre-miRNA is then exported from the nucleus by exportin-5 in a Ran-GTP dependent manner. Once in the cytoplasm, it is processed into a dsRNA of ~22 nt by a complex composed of Dicer and a dsRNA-binding protein (TRBP or PACT). Then, this dsRNA is incorporated into the pre-RISC, where strand selection takes place: one strand is degraded (passenger strand, miRNA*) and the other strand (guide strand, miRNA) remains incorporated in the RISC. Through base pairing, miRNAs can form dsRNA with cellular mRNA, usually at the 3′ UTR region, and may either suppress its translation or induce its degradation. Figure by Wagner Nagib de Souza Birbeire

In canonical biogenesis, miRNAs are transcribed from miR genes as a primary miRNA (pri-miRNA) transcript by RNA polymerase II, the same enzyme that catalyses cellular mRNA transcription. Similar to cellular mRNA, the pri-miRNA has a cap at the 5′ end and a polyadenylated tail at the 3′ end [17]. This first transcript is longer than 70 nt [18] and is frequently several kilobases, bears one or more hairpin structures and can be processed into one or several distinct miRNAs (polycistronic transcription unit) [7] (Fig. 1). This initial transcript is metabolized by a microprocessor complex containing the RNase III Drosha and the co-factor DiGeorge critical region 8 (DGCR8), originating a second transcript named precursor miRNA (pre-miRNA) [19]. This process occurs cotranscriptionally, as evidenced by the processing of miRNAs from unspliced introns [20, 21]. DGCR8 appears to be able to recognize the target RNA, pri-miRNA, through interactions with the ssRNA-dsRNA junction (SD junction) as well as the stem of the hairpin. This interaction directs Drosha activity, enabling the cleavage of the pri-miRNA at an approximately 11 bp site from the SD junction, generating a hairpin structure of approximately 65 nt, pre-miRNA [22–24] (Fig. 1). The pre-miRNA is then recognized and exported from the nucleus by exportin-5, a nucleocytoplasmic transport factor, in a Ran-GTP dependent manner [25]. Once in the cytoplasm, the pre-miRNA has the loop structure removed, releasing a dsRNA of approximately 22 nt. This process is mediated by a complex composed of the RNase III Dicer, which mediates the cleavage, and a dsRNA-binding protein (TRBP or PACT) [7]. The dsRNA is incorporated into the pre-RNA induced silencing complex (pre-RISC), where strand selection based on the thermodynamic properties of the duplex takes place. One strand is degraded in the cytoplasm (passenger strand, miRNA*) and the other strand (guide strand, miRNA) remains incorporated in the RISC, which has a protein from the Argonaute family (Ago1-4) as its core constituent [26]. All Ago proteins, in association with the miRNA guide, can repress mRNA translation [26], but only Ago2 has the ability to cleave the target mRNA in mammals [27]. Through base pairing, miRNAs have the ability to form dsRNA with cellular mRNA and may either suppress its translation or induce its degradation [28]. The interaction of miRNA:mRNA is strongly dependent on nucleotides 2-8 located at the 5′ end of the miRNA; this region is named the seed sequence or seed region [29]. Usually, the seed region binds to the 3′ untranslated region (UTR) of cellular mRNA, but it can also bind to other regions, such as exons [30].

There are some miRNAs that are generated by different routes, in which not all proteins and factors used in the canonical pathway are required. They can be generated by Drosha/DGCR8- or Dicer-independent pathways. These non-canonical Drosha/DGCR8-independent miRNAs can be derived from introns, small nucleolar RNAs (snoRNAs), endogenous short hairpin RNAs (shRNAs) and transfer RNAs (tRNAs) [31].

In vertebrates, despite the many reports of non-canonical miRNA biogenesis pathways, most of the functional miRNAs seem to undergo canonical processing, and only approximately 1 % of conserved microRNAs are produced by Drosha- or Dicer-independent pathways [32].

To distinguish between miRNA and siRNA, we considered that both molecules have interfering roles in gene expression, either through perfect or imperfect base pairing with the mRNA target [33]. Furthermore, in mammalian cells, the RNA interference (RNAi) machinery is shared by the miRNA and siRNA pathways, and there is only one Dicer enzyme [34]. On the other hand, in insect cells, the interfering machinery is separated. In *Drosophila* cells, Dicer-1 is preferentially used to produce miRNA, while Dicer-2 is responsible for generating siRNA [35]. The C6/36 cell line (from *Aedes albopictus* mosquito), which will be referred to at many points in this text, is RNAi defective due to the lack of Dicer-2 activity [36].

### miRNA functions

Among the key roles of miRNAs are gene expression control and mRNA degradation [37]. Complementing their role in gene expression regulation, miRNAs also interact with RNA-binding proteins (RBP), inhibiting the interaction between RBP and mRNA [38]. The classical function of miRNA is post-transcriptional gene expression control; thus, this molecule, besides being involved in the regulation of physiological processes [39], can also be associated with pathological disorders [40, 41]. In addition, miRNAs are present in blood and may be useful as biomarkers for diagnosis and prognosis of several pathologies [42–44]. miRNAs are also involved in somatic cell reprogramming or pluripotent cell maintenance [45], cell cycle control [46], erythropoiesis [47], apoptosis [48, 49], neurogenesis [50], insulin secretion [51], cholesterol metabolism [52], immune responses [53] and viral replication [54]. Interestingly, it has also been shown that miRNAs have roles similar to cellular messengers and signalling molecules [55]. Fabbri et al. [55] showed that specific miRNAs (from tumour cell lines) bind to murine toll-like receptor (TLR)-7 and human TLR-8 in immunological cells, inducing the activation of nuclear factor kappa B (NF-kB) and, consequently, the production of prometastatic inflammatory cytokines. Furthermore, viral miRNAs produced during viral infection may inhibit the viral immune response, which includes inhibiting cytotoxic lymphocyte recognition and modulating the expression of cytokines and chemokines of infected cells [56].

## sfRNA and miRNA in flavivirus infection

Flaviviruses are small spherical viruses of approximately 50 nm with an electron-dense core of approximately 30 nm, surrounded by a lipid envelope composed by two viral proteins: envelope (E) and membrane (M) [57]. The envelope mediates the binding and fusion during viral infection and is the major antigenic determinant, whereas the M protein is a small proteolytic fragment of the prM protein. The flavivirus genome consists of a positive ssRNA containing a cap at its 5′ end but no poly-A tail at its 3′ end. It has a single open reading frame (ORF) flanked by UTRs at its 5′ and 3′ ends [58]. Although both UTRs of flavivirus RNA exhibit great variability, conserved regions and secondary structural conformations are found [59]. Their functions are not completely clear, but they are involved in viral translation, transcription and replication regulation [60, 61]. The UTRs may contain binding sites for viral and cellular proteins and sequences for RNA cyclization [62–64]. Interestingly, flaviviruses transmitted by mosquitoes and ticks have different sequences and secondary structures (hairpin loop) in the 3′ UTR, probably representing an adaptation to the vector [65–67]. The translation of the single ORF of the flavivirus RNA genome produces a large polyprotein cleaved co- and post-translationally by viral and host proteases [68]. The polyprotein is cleaved into 10 proteins, three of which are structural (C, prM and E) and located in the N-terminal region and seven of which are nonstructural proteins (NS): NS1, NS2A, NS2B, NS3, NS4A, NS4B, and NS5 [58]. All these features are shared among the members of the flavivirus genus: Japanese encephalitis virus (JEV), Murray Valley encephalitis virus (MVEV), West Nile virus (WNV), dengue virus (DENV), yellow fever virus (YFV), tick-borne encephalitis virus (TBEV) and Saint Louis encephalitis virus (SLEV), just to mention a few.

In the last few decades, with the development and improvement of molecular biology, microarray and next-generation sequencing techniques, new small non-coding RNAs and miRNAs have been discovered and characterized. The possibility of RNA viruses, which do not possess a nuclear DNA stage in their replication cycle, encoding miRNAs once the first steps in miRNA biogenesis take place in the nucleus has been highly discussed. To investigate this possibility, Rouha et al. [69] modified the TBEV (strain Neudoerfl) genome by genetic engineering. This modification consisted of the insertion of a known heterologous miRNA hairpin precursor from herpesvirus 4 (miR-BART2) in the 3′ UTR of a wild-type TBEV strain. The mutant and wild-type TBEV were introduced into BHK-21 cells by electroporation, and the levels of miR-BART2 were measured by real time PCR and Northern blotting. The results showed an increase in this miRNA 24 and 48 h post infection (hpi). To investigate the role of Drosha, the authors knocked down Drosha protein expression and observed that it was involved in this process. Therefore, this work corroborates the thesis that RNA viruses with an exclusively cytoplasmic replication cycle have the potential to encode functional miRNAs [69]. In support of this idea, Shapiro et al. [70], using a different model (recombinant Sindbis virus producing miRNA), suggested the existence of a cytoplasmic microprocessor of pri-miRNAs and showed that RNA viral infection induced Drosha relocalization to the cytoplasm.

**Fig. 2:**
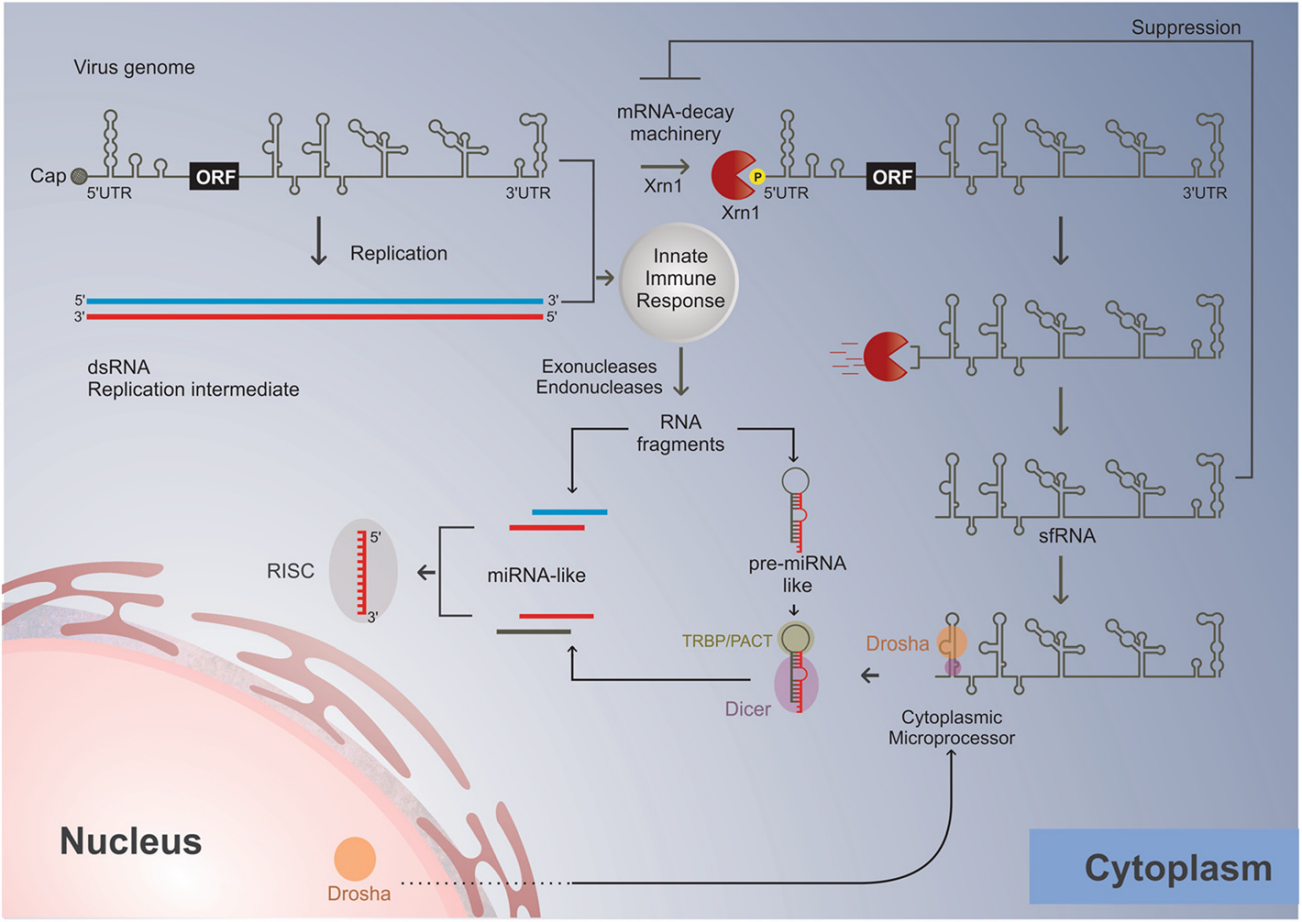
Hypothetical model of flavivirus miRNA-like sRNA biogenesis. Flavivirus genomic RNA is released into the cytoplasm where viral replication and translation take place. In the cytoplasm this RNA may undergo processing by the exonuclease XRN1, which is part of the mRNA decay machinery, and generate the sfRNA. The sfRNA in turn may be further processed into a pre-miRNA-like molecule by a cytoplasmic microprocessor containing Drosha whose relocalization to the cytoplasm may take place during viral infection. The flavivirus genomic RNA or replication intermediates may also be recognized by pattern recognition receptors and activate the innate immune response system. As a consequence, viral genomic RNA can be degraded in several fragments by endo and/or exonucleases. These viral RNA fragments depending on their size and structure may either be processed by Dicer or directly incorporated into RISC. Figure by Wagner Nagib de Souza Birbeire

Viral UTRs have hairpin structures that are believed to act as pre-miRNAs, which can be processed by Dicer or Ago cellular proteins, originating a mature and functional miRNA-like molecule that may contribute to virus and host cell mRNA regulation. Consequently, it has been suggested that viral miRNA biogenesis is Drosha-independent because RNA virus replication takes place in replication complexes located in the cytoplasm, and Drosha is located in the nucleus [15, 16]. RNA viruses are susceptible to degradation by the host RNA decay machinery, and the viral suppression of host exonucleases is crucial for viral pathogenesis [71–74]. In addition, as a consequence of host cell response against the viral infection, several exonucleases and endonucleases are produced to circumvent the accumulation of viral RNAs in the cytoplasm [71]. Therefore, the viral fragments generated by exonucleases, such as subgenomic flavivirus RNAs (sfRNAs) derived from the 3′ UTR, are suggested as one of the sources for viral miRNAs [15, 16] and are required to repress the cellular RNA decay machinery [75–77]. A hypothetical model of flavivirus miRNA-like sRNA biogenesis is presented in the Fig. 2.

To understand the role of viral miRNAs, it is important to briefly introduce the sfRNAs, which are also generated during viral infection. sfRNAs can act as decoy molecules for Dicer and Ago2 [78] or as a source of viral miRNAs [75]. The generation of sfRNAs from flaviviruses was first characterized during Murray Valley viral infections [79] and was later found in Japanese encephalitis virus [80] and West Nile virus [81] infections. However, Pijlman et al. [82] was the first to characterize a host 5′- 3′ exoribonuclease 1 (XRN1) degrading the viral genomic RNA and, consequently, potentially generating sfRNAs (from the 3′ UTR) during West Nile, yellow fever and dengue virus infections. In flavivirus RNA, the 5′ end of the 3′ UTR has conserved secondary structures where the XRN1 stalls, resulting in incomplete degradation of the genomic RNA and generation of the sfRNAs [82, 83]. The sfRNAs, in turn, are able to generate viral miRNA-like molecules through a non-canonical pathway [15, 16]. Further details on the mechanism and structures involved in the generation of sfRNAs can be found at Chapman et al. [84] and Chapman et al. [85].

All available information regarding flavivirus-derived sfRNAs and virus-derived small RNAs (vsRNAs) by the time of submission of this manuscript is summarized in Table 1. In the following sections, more detailed information on West Nile, dengue, Japanese encephalitis, Murray Valley encephalitis and yellow fever viruses will be presented. Experiments that were carried out solely in silico are not included in Table 1.

**Table 1:** Viral sfRNA and small RNA in flavivirus infection.

Virus	sfRNA	Small RNA^a^	Host system	Function	Reference
WNV	600 nt		MEF		Scherbik et al., 2006
WNV	525 nt		BHK-21 and mice	Facilitated viral replication and viral pathogenicity	Pijlman et al., 2008
WNV	525 nt		MEF and mice	Viral evasion	Schuessler et al., 2012
WNV (replicon)			Vero, BHK-21, S2, U4.4 and Ap61	Suppression of RNAi machinery	Schnettler et al., 2012
WNV		19 – 29 nt	*Culex (pipiens) quinquefasciatus*		Brackney et al., 2009
WNV		19, 21 and 28 nt	DC from WT mouse, lymph node and spleen cells from *IFNRab* ^*−/−*^ mice	Possible involvement with IFN response	Parameswaran et al., 2010
WNV		KUN-miR-1 (21 nt)	C6/36, Aag2	Targets GATA4 mRNA	Hussain et al., 2012
DENV-2 (replicon)	400 nt		BHK-21		Pijlman et al., 2008
DENV-1 DENV-2 DENV-3 DENV-4	430 nt 180, 270, 429 nt 410 nt 390 nt		Vero, BHK-21, A549, HepG2, C6/36 and mouse brain	Possible role in viral life cycle and pathogenesis	Liu et al., 2010
DENV-2	429 nt		Vero and BHK-21	Cytopathogenicity and apoptosis	Liu et al., 2014
DENV-2			Huh7	Interference with translation of antiviral interferon-stimulated mRNAs	Bidet et al. 2014
DENV-2			Huh7 and primary monocytes	sfRNA binds TRIM25	Manokaran et al., 2015
DENV-2		17 and 22 nt	Huh.7		Parameswaran et al., 2010
DENV-2		27 nt 21 nt	C6/36 Aag2 and *Aedes aegypti*		Scott et al., 2010
DENV-2		13-19 nt 24–30 nt	*Aedes aegypti*		Hess et al., 2011
DENV-2		vsRNA-1 (22 nt) vsRNA-2 (27 nt) vsRNA-3 (20 nt) vsRNA-4 (21 nt) vsRNA-5 (23 nt) vsRNA-6 (21 nt)	*Aedes aegypti* Aag2, C6/36 and Vero	Regulation of RNA replication by targeting NS1 (vsRNA-5)	Hussain and Asgari, 2014
DENV-4		21 nt 12–36 nt	U4.4 Vero and Huh7		Schirtzinger et al., 2015
JEV	521 - 523 nt		BHK-21 and C6/36	Modulation of viral replication	Lin et al., 2004
JEV			BHK-21	Regulation of viral RNA replication and translation	Fan et al., 2011
JEV			BHK-21, C6/36 and A549	Inhibition of the induction of IFN-beta	Chang et al., 2013
MVEV	600 nt		Brain of infected mice (C3H/HeJ and C3H/RV) and Vero		Urosevic et al., 1997
MVEV	500 nt		BHK-21		Pijlman et al., 2008
YFV	300 nt		BHK-21		Pijlman et al., 2008
YFV	235, 330 and 630 nt 330 nt		BHK-21, Vero, SW13 C6/36		Silva et al., 2010
SRV	500 nt		BHK-21		Pijlman et al., 2008
TBEV		22 nt	IDE8		Schnettler et al., 2014
LGTV		22 nt	IDE8		Schnettler et al., 2014

Grey specifies the molecule found and the respective host system

*Abbreviations*: *DENV* Dengue virus; *JEV* Japanese encephalitis virus; *LGTV* Langat virus; *MVEV* Murray Valley encephalitis virus; *SRV* Saumarez Reef virus; *TBEV* Tick-borne encephalitis virus; *WNV* West Nile virus; *YFV* Yellow fever virus

Host system: A549 (Human lung carcinoma cell line), Aag2 (*Aedes aegypti* cell line), Ap61 (*Aedes pseudoscutellaris* cell line), BHK-21 (Baby hamster kidney fibroblast cell line), C6/36 (*Aedes albopictus* cell line), HepG2 (Human hepatocellular carcinoma cell line*), H*uh7 (Human hepatocarcinoma cell line), IDE8 (*Ixodes scapularis*-derived cell line), MEF (Mouse embryonic fibroblast *cell* line), S2 (*Drosophila melanogaster* Schneider-2 cell line), SW13 (Human adrenal carcinoma cell line*),* U4.4 (*Aedes albopictus* cell line), Vero (Kidney epithelial cells from African green monkey).

^a^All information on small RNAs and miRNAs with either no function described or functionally characterized were included

### West Nile virus

The West Nile virus (WNV) distribution includes temperate and tropical regions of the world. Birds are commonly infected and serve as the prime host reservoir. WNV is transmitted to vertebrates during the female blood meal of various species of *Culex sp.* mosquitoes, e.g., *Culex pipiens*, *Culex tarsalis* and *Culex quinquefasciatus*, depending on the geographical area. Clinical symptoms of WNV infection may include fever, myalgias and meningoencephalitis. WNV infection can progress to a neuroinvasive disease characterized by encephalitis [86].

WNV is one of the most studied flavivirus regarding the generation of sfRNAs, sRNAs and miRNAs. The first report of sfRNAs from WNV infection was during a study on the role of RNase L in the antiviral response to WNV (Eg101 strain). Using MEFs from flavivirus-resistant mice (C3H.PRI-Flv^r^) and susceptible mice (CH3/HeJ) infected with WNV, a band of approximately 600 nt was detected by Northern blot hybridization in both cells. In the absence of RNase L, in MEF cell lines derived from transgenic C57BL/6 RNase L^-/-^ mice, a similar band was also detected. These results indicate that RNase L was not responsible for the generation of the sfRNAs that had been detected [81]. Later, sfRNAs of viral origin were also identified in mammalian cell lines (BHK-21) infected with WNV (Kunjin and NY99 strain), and it was postulated that these non-coding sfRNAs are generated by degradation of the viral genomic RNA by cellular exoribonucleases, such as XRN1. The authors showed that the generation of these sfRNAs in WNV facilitated not only viral replication but also cytopathic effects in cell culture and promoted viral pathogenicity in mice [82]. In addition, Schuessler et al. [87] observed that the synthesis of these sfRNAs by WNV (Kunjin strain) in MEFs and mice might be associated with the escape from the antiviral response mediated by type I interferon (IFN-I). Although the mechanism by which WNV sfRNAs inhibit the function of IFN-I is not completely understood, it has been suggested that sfRNAs directly interact with or antagonize IFN-stimulated gene (ISGs) products (for example, protein kinase R and RNase L) that bind RNAs [87]. Additionally, a suppressor role in the RNAi response to viral infection in mammalian and insect cells has also been ascribed to these sfRNAs [78, 88]. While investigating how the WNV escapes RNA silencing cell defences, Schnettler et al. [88] observed that nonstructural viral proteins (which have dsRNA-binding domain) do not act as RNAi suppressors during WNV (replicon) replication, as observed for other viruses. On the other hand, WNV sfRNA was able to suppress cellular siRNA and miRNA induced in infected cells through competition with Dicer substrates [88].

Parameswaran et al. [89] identified small RNA populations by high-throughput sequencing of six RNA viruses (hepatitis C, polio, dengue, vesicular stomatitis, Flock House and West Nile) during animal cell infection. The abundance and molecular features of vsRNAs (<200 nt) generated by the cell silencing pathway machinery were dependent on the nature of the host and/or virus. The abundance of vsRNAs with regard to host miRNAs was higher with a lack of a functional IFN-α/β receptor in murine cells infected with WNV (NY99-eqhs strain). vsRNAs were also detected in infected dendritic cells from wild-type mice. In these conditions, it was possible to detect three sizes of vsRNAs, 19, 21 and 28 nt. The role or functional activities of vsRNAs were not assessed in this work [89]. Two years later, Hussain et al. [15] reported the generation of miRNA-like sRNAs from WNV using mosquito cells (Aag2 cells, from *Aedes aegypti* and RNAi-defective C6/36, from *Aedes albopictus*) infected with WNV (Kunjin strain). This was the first report demonstrating the generation of functional viral miRNAs during a flavivirus infection. The miRNA discovered by Hussain et al. [15] was named KUN-miR-1 and was found to be derived from the 3′ SL region of WNV RNA. Using a bioinformatics prediction approach, they were able to identify a pre-miRNA hairpin structure at the 3′ UTR of the WNV genome using the VMir software. Based on this prediction, it was possible to design probes for Northern blot analysis, which confirmed the presence of the pre-miRNA (70 nt) and mature miRNA (21 nt) in infected mosquito cells (C6/36 and Aag2). In this model, the Dicer-1 enzyme was shown to be responsible for KUN-miR-1 biogenesis. When the Dicer-1 gene was silenced, KUN-mir-1 production declined, and a significant reduction in WNV replication was also observed. Finally, the function and potential target of KUN-miR-1 in the host cell, in this case Aag2, were identified by a new method where KUN-miR-1 was used as a reverse complementary primer to amplify (RT-PCR) the mRNA to which KUN-miR-1 binds. The alignment of the amplified sequence against the *Aedes aegypti* genome revealed the transcription factor GATA4 mRNA as a target for KUN-miR-1. Evidence shows that KUN-mir-1 increases GATA4 mRNA accumulation in infected cells. It was also shown that the silencing of GATA4 mRNA leads to a decay in WNV RNA, suggesting a role for this gene product in the regulation of viral replication [15].

### Dengue virus

Dengue is an arboviral disease that has a high impact on public health worldwide. It is caused by the four serotypes (DENV1-4) of the virus and is transmitted to humans mainly by the female *Aedes aegypti* infected with DENV. Symptoms during the early stage include fever, malaise, headache, body pain and rash. Dengue fever can progress to severe conditions, such as dengue haemorrhagic fever (DHF) and dengue shock syndrome (DSS) [90].

As previously cited, Pijlman et al. [82] has shown that flaviviruses produce a noncoding RNA nuclease-resistant sfRNA during cell infection. BHK-21 cells infected with a DENV-2 replicon also produced a sfRNA of approximately 400 nt, which correlates with the one of the corresponding 3′ UTR. Several years later, Liu et al. [91] demonstrated the production of sfRNA in several permissive cells (Vero, BHK-21, A549, HepG2, C6/36 cell lines) infected with all four serotypes of dengue (DENV-1, 128 strain; DENV-2, New Guinea C, 43, Fj10 and Fj14 strains; DENV-3, 80 strain; DENV-4, B5 strain). Curiously, the amount of sfRNA produced was not the same for all cells. sfRNA was also identified in brain tissue from mice infected with the different dengue serotypes. Overall, the viral genomic sequence required for sfRNA production is conserved in all serotypes. However, the size of the sfRNAs differed for each serotype: 430 nt for DENV-1, 429 nt for DENV-2 (strain 43 also produced two smaller sfRNAs of 180 and 270 nt), 410 nt for DENV-3 and 390 nt for DENV-4. It is worth noting that these sfRNAs are derived from the 3′ UTR, a region of the viral genome known to be rich in secondary structures and functional RNA elements important for viral replication and translation [91]. The production of a sfRNA of 429 nt in size was reported by Liu et al. [92] in experiments using Vero cells infected with DENV-2 (TSV01 strain). This sfRNA was detected up to 48 hpi in Vero and BHK-21 cells, and the viral RNA structure responsible for the sfRNA generation was identified in the 3′ UTR [92]. These observations matched previous results [91]. Additionally, it was observed that mutations (i.e., deletions) in the sfRNA sequence at the 3′ UTR had no significant effects on viral translation, replication or viral lifecycle. sfRNA production was shown to be required for viral cytotoxicity and apoptosis induction in these infected cell cultures. The mechanism responsible for apoptosis induction in infected mammalian cells was found to be caspase-3-dependent and possibly involves the PI3K-AKT signalling pathway and Bcl-2 as a downstream mediator [92]. In addition, during viral infection, the accumulation of RNA molecules in host cells can activate the RNA decay machinery [72] and trigger an antiviral response [74]. Regarding this subject, Bidet at al. [93] have shown that the sfRNA generated during DENV-2 (New Guinea C strain) infection of a hepatocarcinoma cell line (Huh7) was able to antagonize host RNA-binding proteins (G3BP1, G3BP2 and CAPRIN1) and regulate ISGs expression, consequently avoiding the antiviral IFN-mediated response. Thus, another proposed role for DENV-2 (New Guinea C strain) sfRNAs is the regulation of the host cell antiviral state by circumventing IFN action. In summary, sfRNAs can bypass the host cell response by acting as decoy (or sponge) molecules to the RNA decay machinery, Dicer, factors and proteins involved in immune response and antiviral signalling (such as the RNAi machinery and ISGs mRNA translation) [88, 93, 94]. Recently, Manokaran et al. [95] proposed a model to explain DENV-2 sfRNA-mediated epidemiological fitness. In this study, DENV-2 belonging to different clades (PR-1 and PR-2B) identified in Puerto Rico during the 1994 epidemic were evaluated. In this context, the authors investigated the molecular mechanism behind the PR-2B spread and why this clade replaced PR-1. DENV-2 PR-2B produced increased levels of sfRNA relative to genomic RNA (sfRNA/genomic RNA ratio) compared to DENV-2 PR-1 during viral replication in the Huh7 cell line. The 3′ UTR sequence from DENV-2 PR-2B had three nucleotide substitutions, which contributed to the greater accumulation of sfRNAs compared with PR-1. Manokaran et al. [95] investigated the interaction between host proteins and sfRNAs, employing stable isotope labelling of amino acids in cell culture coupled with quantitative mass spectrometry, and two proteins were identified as significantly enriched: tripartite motif-containing 25 (TRIM25) and mitochondrial antiviral signalling (MAVS). Although both proteins are involved in signalling for IFN expression, when the authors performed an immunoprecipitation to detect TRIM25 or MAVS in infected cells, only sfRNAs from DENV-2 PR-2B were enriched (associated) with TRIM25, confirming the mass spectrometry results. In conclusion, the clade replacement of DENV-2 PR-1 was attributed to the increased sfRNA production and sequence composition of PR-2B, which contributed to circumvent IFN expression [95].

The first report on mammalian host RNAi and miRNA pathway status during dengue virus infection showed that Huh7 infected with DENV-2 (INDI-60 strain) showed a decrease in the mRNA levels of Dicer, Drosha, Ago1 and Ago2 24 and 48 hpi. Consequently, this affected and decreased the host miRNA production. In addition, an important role in the suppression of the RNAi machinery in cells infected with any of the four dengue virus serotypes was attributed to NS4B protein, particularly its transmembrane domain 3 region (TMD3), which interferes with Dicer activity [96]. Employing deep sequencing, Parameswaran et al. [89] identified two vsRNAs (17 and 22 nt) in the Huh7 cell line infected with DENV-2 (16,681 strain). Nevertheless, there was no further information on whether these vsRNAs are miRNAs or siRNAs derived from DENV or their possible function [89]. In the same year, Scott et al. [97] studied the production of DENV-2-specific vsRNAs (Jamaica 1409 strain) in infected mosquitoes and mosquito cells by deep sequencing through analysis of small RNA libraries. The authors investigated the vsRNA profile using *Aedes aegypti* mosquitoes and two different mosquito cell lines, Aag2 cells (RNA interference-competent) and C6/36 (RNA interference-incompetent). The predominant size of DENV-2-specific vsRNAs in *Aedes aegypti* mosquitoes and the Aag2 cell library was 21 nt, and in the C6/36 cell library, it was 27 nt. In vitro studies demonstrated that vsRNA production in C6/36 cells led to inefficient Dicer-2 cleavage of long dsRNAs. These data suggest that the lack of a complete and functional RNAi pathway in C6/36 cells may favour arbovirus replication [97]. Considering the importance of sRNAs in antiviral defence in mosquito cells, Hess et al. [98] determined the vsRNA profile in *Aedes aegypti* mosquitoes infected with DENV-2 (Jamaica 1409 strain). Applying the deep sequencing technique, the vsRNAs (20–23 nt) that had been previously reported by Scott et al. [97] were confirmed. In addition, unusually small RNAs (usRNA, 13–19 nt) and sRNAs presenting a size (24–30 nt) consistent with PIWI pathway small RNAs were also identified. These results suggest a role for the PIWI pathway in the antiviral response in *Aedes aegypti* [98].

In an attempt to elucidate the relationship between the IFN response and the RNAi machinery, deep sequencing of mosquito (U4.4) and primate (Vero and Huh7) cell lines infected with DENV-4 was carried out. As initially expected, because RNAi is the major antiviral response in insect cells, a much higher production of vsRNAs was observed in U4.4 than Vero or Huh7 cells. Two different mammalian cell lines, one capable of mounting IFN-I responses (Huh7) and the other not capable (Vero), were used to investigate the effect of IFN responses on the vsRNA production in DENV-infected cells. It was expected that DENV-4-infected Vero cells would show a more extensive repertoire of vsRNAs than DENV-4-infected Huh7; however, a significantly greater percentage of vsRNAs were observed in Huh7 cells. vsRNAs from DENV-4-infected U4.4 cells were mostly 21 nt and originated in a similar proportion from the genome and antigenome. In the mammalian cells, vsRNAs ranged from 12–36 nt with a modest peak at 24 nt, and most were derived from the positive-sense strand. When the DENV-4 genome was mapped in search of targets for the 21 nt vsRNAs identified in DENV-4-infected Huh7 cells, a major peak was observed at the 5′ end of NS5. In the case of DENV-4-infected Vero cells, additional peaks were identified at the 5′ and 3′ ends of the E gene and at the 3′ end of NS5. All the peaks observed in the mammalian cells could also be found in the mosquito cells, although not always with corresponding intensities. According to Schirtzinger et al. [99], the characteristics of the vsRNAs found in the mammalian cells broad size distribution and origin mostly on the positive-sense strand suggest they are not Dicer products [99].

Recently, two studies reported conflicting results regarding the existence of miRNAs derived from the DENV genome. The first study investigated the 5′ UTR and 3′ UTR from the genome of DENV-2 (New Guinea strain) as candidate source sequences of viral miRNAs, which are known for presenting stem-loop structures. These structures could be potential targets for enzymes belonging to the eukaryotic RNA silencing machinery. In this work, the miRNA analysis by deep sequencing was first evaluated in infected *Aedes aegypti* mosquitoes. RNAfold software was used for the prediction of precursor stem-loop structures in the 5′ and 3′ UTRs of the viral genome, which, together with the deep sequencing, allowed the selection of six different miRNA-like molecules (vsRNA1-6) for further analysis. After the infection of Aag2 cells with DENV-2 and treatment with a synthetic inhibitor specific to the vsRNA-5, one of the selected vsRNA candidates, the authors observed a significant increase in viral replication. The same results were observed in C6/36 and Vero cell lines. vsRNA-5 was localized to the 5′ portion of the 3′ UTR, the main protein involved in vsRNA-5 biogenesis in insect cells was Ago2 and the DENV-2 NS1 gene was identified as the vsRNA-5 possible target. The proposed role of vsRNA-5 is the down-regulation of viral RNA replication in this DENV-2 model [16]. On the other hand, Bogerd et al. [100] reported that the infection of human cells (Huh7) with DENV-4 (TVP 360 strain) or WNV does not produce viral miRNAs. A sRNA library from DENV-4-infected Huh7 cells did not show enrichment for any particular length, such as the 22 ± 2 nt sizes predicted for Dicer cleavage products. However, it is important to note that when human Dicer-deficient cells generated from the 293T cell line (NoDice) were infected with DENV-4, the virus replication proceeded “slightly more slowly” compared with wild-type 293T cells. In regard to yellow fever and WNV infections in NoDice and normal 293T cell lines, no relevant difference in viral replication was found. As the majority of vsRNAs detected in this study originate from the positive RNA strand, these authors suggest that they were the result of viral genome breakdown [100].

Because the methodologies used to predict plant and animal miRNAs cannot be accurately applied to viral miRNAs, Ospina-Bedoya et al. [101] designed a different approach to predict vsRNAs derived from the dengue virus genome. This strategy involved scanning the genome in search of pre-miRNAs using the VMir software, filtering these results with the ncRNA feature extraction and pre-miRNA classification web tool, validating the secondary structure of the pre-miRNAs using Mfold and, at last, identifying mature miRNAs with the MaturePred tool. Applying this methodology to the DENV-2 genome (GenBank accession numbers: JF357906.1 and FJ390389.1), they were able to identify eight putative miRNAs, all derived from the negative strand. These miRNAs are scattered throughout the viral genome. The authors also note that most of these miRNAs do not start with a U at their 5′ end as would be expected of miRNAs recognized by Ago1, which may indicate a non-canonical pathway origin. Despite not carrying out any in vitro or in vivo validation of the identified miRNAs, the analysis included a final step in which the MiRanda software was used to search the human genome UTRs for functional targets. The result was the identification of 53 target transcripts for these eight miRNAs, suggesting that most of them have more than one target. A functional enrichment analysis of these potential targets showed the most enriched functional clusters to be anatomical structure formation involved in morphogenesis and cell projection morphogenesis [101]. It is worth noting that the in silico predicted miRNAs do not match those identified in vitro by Hussain and Asgari [16]. Although both used the DENV-2 New Guinea C strain, the results are quite different.

### Japanese encephalitis virus

Japanese encephalitis virus (JEV) is most prevalent in temperate and tropical areas of eastern and southern Asia. The virus transmission cycles involves wild birds and domestic pigs as reservoirs. Among the major vectors of JEV are the mosquitoes *Culex tritaeniorhynchus*. The transmission to humans may cause symptoms such as lethal meningomyeloencephalitis, but most human infections are asymptomatic or induce influenza-like symptoms as a mild febrile illness [102].

Lin et al. [80] characterized a specific plus-strand RNA molecule of 521 to 523 nt in mammalian (BHK-21) and mosquito (C6/36) cell lines infected with nine strains of JEV (RP-2, RP-9, TP-53, CH-1392, CC-27, S-26, PC-806, Beijing-1 and Nakayama). However, this molecule accumulates more abundantly in mosquito cells than in mammalian cells. This sfRNA, called a small RNA by these authors, was found to be identical to the highly conserved sequence at the 3′-terminal region of the 3′ UTR of all JEV strains. The authors speculated that the transcription of JEV minus-strand RNA by an internal promoter can generate the plus-strand JEV sfRNA. Alternatively, the sfRNA molecule may also be a product of viral genome cleavage by host RNases [80]. In a subsequent work, the same group revealed preliminary evidence suggesting that JEV sfRNA (RP-9 strain) could modulate viral replication and translation. JEV sfRNAs were detected from 28 to 48 hpi in infected BHK-21 cells. When sfRNA generation and accumulation (evaluated by Northern blot) were compared with viral genomic RNA and antigenomic RNA amounts (determined by qPCR), the authors observed that sfRNA appearance was correlated with the antigenome RNA plateau (from 32 to 40 hpi). This suggests that sfRNA may regulate antigenome synthesis. To investigate the role of sfRNA in JEV replication, plus- or minus-strand forms of the sfRNA were transfected into JEV-infected cells. The presence of minus-strand JEV sfRNA, *in trans*, in infected BHK-21 cells increased antigenome JEV RNA synthesis. Thus, this result suggests that *in trans* minus-strand sfRNA inhibited the JEV-derived plus-strand sfRNA by complementarity. Consequently, it can be concluded that sfRNA controls antigenome RNA synthesis. When the viral translation was evaluated (by a *Renilla* luciferase assay employing a JEV minicon construction containing a *Renilla* luciferase reporter gene) the addition of both, minus- or plus-strand sfRNAs inhibited the luciferase translation [103]. Furthermore, Chang et al. [104] showed that the ratio between JEV sfRNA and genomic RNA (sfRNA:gRNA) was increased in a later stage (5 dpi) compared with acute JEV (RP-9 strain) infection (48 hpi) in BHK-21 and C6/36 cell lines (IFN-incompetent cells). When a JEV-infected IFN-competent lung carcinoma cell line (A549) was transfected with sfRNA, the expression of IFN-β decreased significantly. The IFN-β inhibition was possibly due to the role of sfRNA (dose-dependent response) in preventing the phosphorylation of the interferon regulatory factor-3 (IRF-3) transcription factor and its nuclear translocation. In addition, JEV-infected cells transfected with sfRNA had reduced apoptosis compared with JEV-infected and untransfected cells. Together, these effects contributed synergistically to viral infection maintenance. Thus, JEV sfRNA is able to block the host antiviral response [104].

### Murray Valley encephalitis virus

Murray Valley encephalitis virus (MVEV) is endemic to Australia. Water birds are the main reservoir for this virus, and the major mosquito vector is *Culex annulirostris*. Human infection occurs through bites from infected mosquitoes. Some people may experience a mild form of the disease with symptoms such as fever, headaches, nausea, vomiting, diarrhoea, macular rash and cough. Neurological features may occur, but the majority of MVEV infections are sub-clinical and do not produce disease symptoms [105, 106].

The first detection of a MVEV sfRNA of 600 nt was reported by Urosevic et al. [79] in the brains of C3H/HeJ (flavivirus susceptible) and C3H/RV (flavivirus resistant) infected mice (MVEV OR2 strain) at 3, 4 and 6 dpi. MVEV sfRNA was also found in infected Vero cells. Although the sfRNA hybridized with a fragment from a MVEV cDNA clone, which corresponds to the 3′ terminal region of the MVEV genome, the origin and role of this RNA in viral replication were not defined [79]. Later, Pijlman et al. [82] demonstrated the sfRNA production in cell lines infected with several flaviviruses, among which were MVEV and Alfuy virus (a subtype of MVEV). Specifically, BHK-21 cells infected with MVEV and Alfuy virus had sfRNA species of approximately 500 nt. Therefore, independent of cell source, MVEV infection can produce sfRNAs. However, the role of these molecules in MVEV replication requires further exploration.

### Yellow fever virus

Yellow fever virus (YFV) is the aetiological agent of a viral haemorrhagic fever transmitted by *Aedes aegypti* mosquitoes in Africa and Central and South America. YFV infection causes significant morbidity and mortality in Africa. The clinical spectrum of yellow fever ranges from subclinical infection to systemic disease. The acute infection may induce high fever, chills, intense headache, malaise, generalized myalgia, nausea and vomiting [107, 108].

The first report of sfRNA generation during YFV infection was described by Pijlman et al. [82]. In BHK-21 cells infected with YFV, a sfRNA with approximately 300 nt derived from the 3′ UTR was first identified by Northern blot hybridization. Later, Silva et al. [109] described the molecular mechanism behind YFV sfRNA production. In mammalian cell lines (BHK-21, Vero E6, SW13), YFV (YFV-17D strain) infection generated three sfRNAs termed RNA A (630 nt), RNA B (330 nt, also called sfRNA1) and RNA C (235 nt, also called sfRNA2), but in YFV-infected mosquito cells (C6/36), only sfRNA1 was detected. To investigate the role of XRN1 in the sfRNA production, YFV-infected SW13 cells were transduced with lentiviruses expressing shRNA to silence XRN1 transcripts. The production of sfRNA after XRN1 silencing was reduced approximately 90 %. These results were confirmed in vitro by incubating purified XRN1 with the YFV template. The viral RNA structure required for sfRNA generation was identified by disrupting the SL-E region in the 3′ UTR of YFV RNA because the 5′ end of sfRNA1 and sfRNA2 are located a few nucleotides upstream of this region. When mutations were introduced in SL-E region, which is part of a pseudoknot structure, no sfRNA was detected in BHK-21 cells infected with the YFV mutants. Thus, in both mammalian and insect cells, a pseudoknot RNA structure in the 3′ UTR of the YFV genome is essential for XRN1 stalling and sfRNA generation [109].

## Further considerations on flavivirus-derived sfRNAs and miRNAs

Pijlman et al. [82] identified the production of sfRNA species as a unique feature of the flavivirus genus. Production of sfRNA was not observed during the infection of a virus belonging to another genus of the *Flaviviridae* family [82], although a similar mechanism based on the stalling of XRN1 was proposed to be involved in the generation of much longer non-coding RNAs (~9.6–12.5 kb) in the case of hepatitis C and bovine viral diarrhoea viruses [77]. Another flavivirus investigated by Pijlman et al. [82] was Saumarez Reef virus (SRV), which is transmitted by ticks but cannot induce encephalitis. SRV also produces a sfRNA of approximately 500 nt, but the role of this molecule in SRV replication was not investigated [82]. In fact, all members of the flavivirus genus that have been investigated to date regarding the production of sfRNAs during infection (DENV, YFV, MVEV, WNV, JEV, SRV, TBEV, Modoc virus, Apoi virus, Rio Bravo virus, Montana myotis leukoencephalitis virus, Yokose virus, cell-fusing agent virus, Culex flavivirus) have shown positive results [75, 110], despite the fact that not all flaviviruses present the same secondary structures in the 3′ UTR [111].

vsRNAs have also been identified in tick-borne flaviviruses. To elucidate the antiviral role of RNAi in ticks, Schnettler et al. [112] studied the vsRNA profile of an *Ixodes scapularis*-derived cell line (IDE8) either infected with a tick-borne flavivirus (TBEV or Langat virus) or transfected with the corresponding replicon. In both cases, vsRNAs were for the most part 22 nt long, scattered throughout the genome and mapped in similar proportion to the sense and antisense strands. Additionally, a higher frequency of vsRNAs was derived from the 5′ and 3′ ends of the genome. The knockdown of genes identified by phylogenetic analysis as homologous to either Dicer-2 or Ago2 followed by either Langat virus infection or its replicon transfection allowed the identification of Ago30 and Ago16 as mediators of antiviral activity against this virus. In the same work, an inhibitory effect of the sfRNA on the RNAi machinery was also reported [112].

In addition to the ones that have been previously cited, many in silico predicted flavivirus-derived miRNAs can be found in the Vir-Mir database. The methodology used to build up the Vir-Mir database was based on the prediction of candidate hairpin structures compatible with the characteristics of known pre-miRNAs from the genomic sequences of 2266 viruses available at the time in the GenBank database. Afterwards, additional filters also based on the characteristics of known miRNAs, such as GC content, core minimum free energy (MFE), hairpin MFE and ratio of core MFE to hairpin MFE, were applied to reduce the number of false positives [113]. In total, the Vir-Mir database lists 171 flavivirus-derived miRNA candidates that have been predicted using this strategy [114].

Furthermore, a study by Brackney et al. [115] notes the role of RNAi in WNV genetic diversification in infected *Culex (pipiens) quinquefasciatus* mosquitoes. The midgut of infected mosquitoes (NY99 strain) collected at 7 and 14 days post infection (dpi) was used in the construction of a small RNA library. The size of the sRNAs, which mapped to WNV genome, ranged from 19 to 29 nt, but the most abundant sizes were 20, 21 and 22 nt. Considering that the RNAi machinery is sequence-specific, and mutations in the target dsRNA sequence can compromise its efficiency, the target regions of RNAi in the WNV genome were evaluated. Mutations were detected in both viral targets: NS5 (7 and 14 dpi samples) and 3′ UTR (14 dpi samples). In conclusion, the authors suggest a correlation between RNAi machinery activity and viral evolution (positive selection), which reinforces the importance of the mosquito to WNV diversification [115].

As a final remark, it is important to remember that the different flaviviruses present variations in their 3′ UTRs, such as deletions and/or duplications of conserved structures. Just to cite an example, Bryant et al. [116] reported the existence of a 16 nt duplication, which created in tandem repetition of a conserved hairpin, stem-loop and pseudoknot RNA structure, in 19 YFV isolates from South America [116]. Taking this into consideration and the fact that sfRNAs may be the source of viral miRNAs by a non-canonical pathway, as previously suggested by Hussain et al. [15] and Roby et al. [75], variations in the 3′ UTR may have an impact on the different expression patterns of non-coding RNAs observed for the different viruses.

## Human and murine miRNAs modulated during flavivirus infection

Recently, several studies have shown an intimate relationship between RNA virus infection, miRNAs and host response [117, 118]. During viral infection, cells can alter the miRNA profile involved in physiological functions and establish an antiviral state. Human miRNAs have been described as regulators of gene expression in viral replication and host immune responses [119]. For this topic, all available information on human and murine miRNAs produced in response to flavivirus infection was gathered. However, only the miRNAs with confirmed function through biological assays, such as the use of mimic miRNAs, miRNAs inhibitors and/or luciferase reporter vectors, are presented in Table 2. The use of an asterisk to identify the passenger strand has been replaced in miRBase by the use of either -5p or -3p which identify the arm of the hairpin precursor from which the mature miRNA originates. However, the nomenclatures seen in the text follow the information as presented in the original articles.

**Table 2:** Function of human and murine miRNAs modulated during flavivirus infection

Flavivirus	miRNA	Host system	Function	Reference
WNV	Hs_154	HEK293, SK-N-MC, Huh7, Huh7.5 and mouse brain	Modulates the apoptotic response	Smith et al., [120]
WNV	miR-532-5p	HEK293 and mouse brain	Suppresses genes SESTD1 and TAB3	Slonchak et al., [121]
DENV-2	miR-146a	PBMC and THP-1	Dampens IFN-β production by targeting TRAF6	Wu et al., [118]
DENV-1, 2 and 3	miR-30e*	U937, HeLa and PBMC	Up-regulates IFN-β and ISG expression	Zhu et al., [123]
DENV-2	miR-150	PMBC	Regulates SOCS1 expression	Chen et al., [126]
DEN-2	miR-223	EAhy926, HepG2 and Vero	Represses STMN1	Wu et al., [127]
DENV-2 and 4	Let-7c	Huh7	Modulates BACH1 and HO-1 expression	Escalera-Cueto et al., [124]
JEV	miR-29b	BV-2 and primary microglial cells	Microglia activation by targeting TNFAIP3	Thounaojam et al., [130]
JEV	miR-155	BV-2, mouse primary microglial cells, mouse and human brains	Inhibits SHIP1 expression and promotes IFN-β expression	Thounaojam et al., [131]
JEV	miR-155	CHME3	Represses IFN-β production	Pareek et al., [132]
JEV	miR-15b	BV-2, U251, HeLa and mouse brain	Represses RNF125, a RIG-I inhibitor	Wu et al., [127]
JEV	miR-146a	CHME3	Down-regulation of TRAF6 expression	Sharma et al., [133]

Abbreviations: *BACH1* BTB and CNC homology 1, basic leucine zipper transcription factor 1, *DENV* dengue virus, *HO-1* haeme oxygenase-1, *IFN* interferon, *JEV* Japanese encephalitis virus, *RIG-I* retinoic acid-inducible gene 1, *RNF125* ring finger protein 125, *SESTD1* SEC14 and spectrin domains 1, *SHIP1* Src homology 2-containing inositol phosphatase 1, *SOCS1* suppressor of cytokine signalling 1, *STMN1* stathmin 1, *TAB3* TGF-beta activated kinase 1/MAP3K7 binding protein 3, *TNFAIP3* tumour necrosis factor alpha-induced protein 3, *TRAF6* TNF receptor-associated factor 6, *WNV* West Nile virus

Host system: BV-2 (Mouse microglial cell line), CHME3 (Human brain microglial cell line), EAhy926 (Human vascular endothelial cell line), HEK293 (Human embryonic kidney 293 cell line), HeLa (Human cervical carcinoma cell line), HepG2 (Human hepatocellular carcinoma cell line), Huh7 and Huh7.5 (Human hepatocarcinoma cell line), PBMC (Human peripheral blood mononuclear cells), SK-N-MC (Human neuroblastoma cell line), THP-1 (Human monocytic cell line), U251 cells (Human astrocytoma cell line), U937 (Human leukaemic monocyte lymphoma cell line) and Vero (African green monkey kidney cell line)

### West Nile virus

In the case of WNV (385-99 strain) infection, many different cellular miRNAs have been identified as differentially expressed in infected cells (HEK293 and SK-N-MC cell lines) compared with uninfected controls, using miRNA-specific microarrays. Interestingly, the profile of differentially expressed miRNAs in infected cells and uninfected cells treated with interferon is quite different, which indicates that the differentially expressed miRNAs are not simply a result of the interferon-induced antiviral response. The most highly expressed miRNA, Hs_154, identified in this study was shown to be consistently up-regulated independent of the infected cell line (HEK293, SK-N-MC, Huh7, Huh7.5) and in the brains of infected mice at 11 dpi, temporally correlating with pathogenesis. Cells transfected with a mimic of the mature dsRNA and subsequently infected with WNV had lower viral titres at 2 and 3 dpi, suggesting an antiviral role for Hs_154. A time-course experiment that was carried out in HEK293 cells infected with WNV showed that Hs_154 up-regulation could be detected at 36 hpi and was stronger at 48 hpi, when strong cytopathic effect associated with apoptosis can already be observed, suggesting that its induction occurs in response to viral infection. However, treating cells with different inducers of antiviral pathways, such as poly I:C, IFN or another flavivirus (DENV), did not induce Hs_154 miRNA expression. To identify Hs_154 miRNA targets, RISC immunoprecipitation was carried out followed by a search for a match in the 3′ UTR of the enriched mRNAs to the seed sequence of Hs_154. Among the identified potential targets, two were selected for further analysis: CCCTC binding factor (CTCF) and EGFR-coamplified and overexpressed protein (ECOP). The 3′ UTRs of these mRNAs were inserted downstream of a luciferase reporter vector and transfected into cells followed by WNV infection or the concomitant transfection of a mimic of Hs_154 miRNA. In both cases, WNV infection or Hs_154 mimic co-transfection, a decrease in the expression of luciferase was observed, which demonstrates these mRNAs are targeted by the Hs_154 miRNA. Additionally, the infection of cells that had been transfected with either CTCF- or ECOP-expressing vectors resulted in a decrease in the cleavage of poly (ADP-ribose) polymerase, a substrate for caspase, and also in TUNEL staining. The same results were observed when cells were transfected with an inhibitor of Hs_154 miRNA. As both proteins are known to possess antiapoptotic activity, and given the results obtained, their targeting by Hs_154 miRNA can be assumed to play an important role in the apoptosis observed during viral infection. The authors conclude that although the inhibitory effect of Hs_154 miRNA on viral replication implies an antiviral host response, its association with apoptosis, mainly when considering infection of the central nervous system, may represent an adverse side effect for the host [120].

Regarding the host miRNA antiviral response, Slonchak et al. [121] evaluated WNV (Kunjin strain) infection in mammalian cells (HEK293) and mice. miRNA expression analysis by RNAseq, qRT-PCR, and Northern blot revealed that three host miRNAs were significantly up-regulated during infection (miR-1271-5p, miR-532-5p and miR-1307-3p). To investigate the role of these three miRNAs in WNV replication, HEK293 cells were first transfected with miRNA-specific mimics, nonspecific mimics or inhibitors and then infected. Only in the cells transfected with the miR-532-5p mimic was viral titre significantly reduced. Opposing results were observed when the cells were transfected with the miR-532-5p inhibitor. The interaction between miR-532-5p and a putative site in the WNV RNA genome was investigated through computational screening using the RNAhybrid software. Although this analysis predicted two potential binding sites, which were experimentally assessed by a luciferase reporter assay, no functional effect on expression of luciferase was observed. These results suggested that miR-532-5p has a host target. To identify possible miR-532-5p targets in human genes, the whole genome was evaluated by computational prediction assessing several features, such as evolutionary conservation of sites (miRDB, TargetScanHuman), seed region complementarity (Diana Micro-T, micrroRNA.org) and thermodynamics of duplexes (TargetMiner, RNA22). Ten candidates (CPEB3, FAM116A, SESTD1, RASSF5, GBP1, ZNF536, RAB11A, ARMC8, SMAD2 and TAB3) were selected based on simultaneous prediction by at least 5 different algorithms. To validate the predicted targets, they were evaluated in WNV-infected and uninfected HEK293 cells using qRT-PCR. The mRNA levels of TGF-β activated kinase 1/MAP3K7 binding protein 3 (TAB3) and SEC14 and spectrin domains 1 (SESTD1) decreased significantly in WNV infection. SESTD1 is important for cellular Ca^2+^ influx, and TAB3 is required to NF-kB signalling. The interaction between miR-532-5p and its targets (TAB3 and SESTD1) was further validated by Western blot, use of miR-532-5p mimics and luciferase assays. Similar to the qRT-PCR results, TAB3 and SESTD1 protein expression was significantly decreased during infection. Similar results were observed in non-infected cells treated with a miR-532-5p mimic. Finally, the luciferase reporter assays confirmed targeting of the TAB3 and SESTD1 3′ UTRs by miR-532-5p. In conclusion, these results indicate that WNV infection induces cellular miR-532-5p, which in turn affects the host antiviral response [121].

Concerning WNV infection (NY99 strain), Kumar and Nerurkar [122] examined the miRNA expression in the brains of infected mice at the peak viral load (8 dpi) using a qPCR array. Compared with mock-infected mice, 36 miRNAs were up-regulated and 103 down-regulated by at least 2-fold. Some presented a fold change higher than 10. The up-regulated miRNAs include let-7a and miR-99a and the down-regulated include miR-196a, miR-470, miR-21*, miR-208a, miR-683, miR-184, miR-693-3p, miR-202-3p, miR-429, miR-878-3p and miR-327. Many of the miRNAs that were identified as differentially expressed were associated with neurodegenerative diseases (Parkinson’s, Alzheimer’s and Huntington’s diseases) and other virus-mediated neurological diseases, suggesting the modulation of common neurological pathways. Target prediction and analysis of the miRNA-mediated networks indicated cell death and survival, immune-cell trafficking, cell-mediated immune response and inflammatory response as the leading pathways associated with the modulation observed during infection. Because all these pathways are associated with WNV pathogenesis in this animal model, the identified miRNAs may play significant roles during viral infection. Additionally, when the mRNA expression profile was analysed, an inverse correlation between the modulated miRNAs and target neuroinflammatory genes was found, supporting a role for them in disease pathogenesis [122].

### Dengue virus

Up-regulation of human miR-146a was found in human primary monocytes and THP-1 monocytes infected with DENV-1 (Hawaii strain), DENV-2 (New Guinea C strain), or DENV-3 (H241 strain). Investigating the role of miR-146a in DENV-2 infection, Wu et al. [118] observed that this miRNA promoted viral replication even at different multiplicities of infection (MOI). The molecular mechanism behind the proviral role of miR-146a involves the down-regulation of IFN-β through targeting of TNF receptor associated factor 6 (TRAF6), an adaptor in the IFN pathway signalling. This was the first study that showed the role for a host miRNA in DENV infection [118].

Similarly, taking into account that miR-30e* promotes NF-kB activation, which is essential for antiviral innate immune activation, Zhu et al. [123] investigated its role in DENV infection. HeLa cells were infected with DENV-1 (Hawaii strain), DENV-2 (New Guinea C strain), or DENV-3 (H241 strain). Six hours after infection, the relative miR-30e* expression was significantly increased in infected cells regardless of serotype. Similar results were observed in U937 cells and PBMCs infected with DENV. When a miR-30e* mimic was transfected into HeLa, U937 and PBMC cells followed by DENV-2 infection, viral replication was suppressed. On the other hand, IFN-β mRNA expression and IFN-β secretion in the cell culture supernatant was increased. Additionally, DENV-2 replication was rescued when miR-30e* was silenced. Taking into account the association between IFN-β production and NF-kB activation during viral infection, the effect of miR-30e* on the gene expression of NF-kB pathway components was investigated. A luciferase reporter plasmid construction carrying the 3′ UTR sequence of IkBα (nuclear factor of kappa light polypeptide gene enhancer in B-cells inhibitor, alpha) mRNA (an inhibitor of NF-kB) was transfected into HeLa and U937 cells. When these cells were co-transfected with a miR-30e* mimic, the luciferase activity was suppressed. In conclusion, during DENV infection, miR-30e* is an important regulator of antiviral response inhibiting virus replication [123].

Furthermore, to investigate the human miRNA modulation during DENV infection, Huh7 cells were infected with DENV-2 (New Guinea C strain), and the miRNA profile was analysed with a GeneChip miRNA Array. From 846 human miRNAs, nine (let-7c, miR-92b*, miR-130b, miR-140-3p, miR-149*, miR-181c*, miR-374a*, miR-453, miR-1268) were identified as differentially expressed. Several targets could be predicted in the DENV-2 genome for the differentially expressed miRNAs, although the miRNA with the highest scoring targets was let-7c. Moreover, it was overexpressed at different times post infection (12, 24 and 48 hpi) in Huh7 as well as in U937-DC-SIGN cells. The predicted target for let-7c in the viral genome was NS1 (which is highly conserved in different DENV-2 strains) and NS4B (which is not conserved). However, when Huh7 cells were pretreated with a let-7c mimic and then infected with DENV-2 or DENV-4, viral replication and NS1 protein production were significantly reduced. The opposite was observed when let-7c was inhibited. Curiously, no targets for let-7c were found in the DENV-4 (V3361-1956 strain) genome. These results suggested that the let-7c target may be a human mRNA involved in antiviral response. TargetScan identified BACH1, a transcription inhibitor, as one of the genes predicted to have target sequences for let-7c. Experiments employing let-7c mimics or let-7c inhibitor confirmed BACH1 as a let-7c target; their expression levels were shown to be negatively correlated. As a consequence of BACH1 down-regulation by let-7c, the heme oxygenase-1 gene may be overexpressed, contributing to the development of an anti-inflammatory microenvironment, which may antagonize the oxidative stress response induced by the infection [124]. It is worth noting that in WNV infections, down-regulation of let-7c expression was observed in the brains of infected mice [122].

One of the features of DHF/DSS is the occurrence of a cytokine storm, which leads to serious plasma leakage. Two studies have investigated this feature with regard to the human miRNA profile induced by DENV infection [125, 126]. In the first, Qi et al. [125] investigated whether the excessive secretion of cytokines during DENV-2 infection is a result of miRNA activity on cytokine genes or regulators. To answer this question, PBMCs from healthy donors were infected with DENV-2 (New Guinea strain). The cytokines that were shown to be most highly expressed in infected cells were CCL5, IL-6 and IL-8 (6 and 12 hpi). The expression profile of infected and uninfected cells was evaluated using a miRNA microarray, and 16 miRNAs were reported to be up-regulated (miR-4290, miR-4279, miR-625*, miR-let-7e, miR-1290, miR-33a, miR-3686, miR-378, miR-1246, miR-767-5p, miR-320c, miR-720, miR-491-3p, miR-3647, miR-451 and miR-4286) and 4 down-regulated (miR-106b, miR-20a, miR-30b and miR-3653) during dengue infection. The differential expression of most of the miRNAs identified in the microarray analysis was confirmed by qRT-PCR, but none of them were evaluated for function. Three different online databases (Sanger miRBase, miRanda and TargetScan) were used to predict target genes for the differentially expressed miRNAs. Common targets identified by the different databases were submitted to ingenuity pathway analysis software, and several cell processes were identified to be associated with the predicted targets, such as signal transduction, cell response to stimulus, biological regulation, and metabolism. Finally, to evaluate the relationship between cytokine expression and miRNA profile, a sequence homology search was carried out between the mRNAs of cytokine genes and the differentially expressed miRNAs. This analysis identified IL-6 and CCL3 as potential targets of miR-let-7e and MIF, CCL5 and CXCL1 as potential targets of miR-451, miR-106b and miR-4279, respectively. The lower levels of miR-106b and the higher levels of expression of CCL5 are compatible with a derepression mechanism. However, this mechanism is not able to explain the high levels of miR-let-7e and its target cytokine genes (CCL3 and IL-6). The authors also identified epigenetic regulators as the potential targets of miR-let-7e (EZH2) and miR-30b (methyltransferase 3A). Therefore, they hypothesize that the high levels of some cytokines may be a consequence of targeting the elements involved in epigenetic processes [125]. A report by Chen et al. [126] further investigated the relationship between cytokine and miRNA expression during DENV infection by focusing on suppressor of cytokine signalling 1 (SOCS1), a negative regulator of several cytokines. In this case, however, the role of DENV-induced human miRNA in the regulation of SOCS1 expression in DF and DHF patients was investigated. Significantly higher SOCS1 mRNA was observed in leukocytes from DF compared with DHF patients. Additionally, when PBMCs from healthy donors were infected with DENV-2 (New Guinea strain), SOCS1 mRNA expression was shown to be increased in DENV-2-infected cells compared with mock-infected cells, especially in CD14^+^ cells (monocytes and macrophages). A bioinformatics search for miRNAs that target the SOCS1 3′ UTR resulted in 11 candidates (miR-15a, miR-20, miR-21, miR-96, miR-126, miR-146, miR-150, miR-181a, miR-155, miR-221 and miR-57). All of them were evaluated by qRT-PCR, but only miR-150 was up-regulated in DHF samples. Interestingly, SOCS1 mRNA expression in leukocytes from DHF and DF patients was inversely correlated with miR-150 expression. In the DHF patients, SOCS1 expression was down-regulated and miR-150 was up-regulated, and the inverse was observed in DF samples. To confirm the association between SOCS1 and miR-150 expression, PBMCs from healthy donors were infected with DENV-2 and then transfected with miR-150 mimics. As a result, the SOCS1 mRNA expression was significantly repressed. In conclusion, the authors showed for the first time that the pathogenesis of DHF is associated with the repression of SOCS1 concomitant to the increased expression of miR-150 [126].

Evaluating the miRNA profile in a human vascular endothelial cell line (EAhy926) infected with DENV-2 (TR1751 strain) by microarray and qRT-PCR, 12 miRNA candidates were found to be up-regulated (miR-3178, miR-324-3p, miR-937, miR-3195, hsv1-miR-H6-3p, kshv-miR-K12-12*, let-7a-2*, miR-20b*) and down-regulated (miR-21*, miR-2116, miR-142-3p, miR-223). Among the down-regulated candidates, miR-223 showed a 30 % reduction when compared to uninfected cells. Similar results were observed in other mammalian cells (HepG2 and Vero). To investigate the involvement of miR-223 in DENV-2 replication, this miRNA was overexpressed or reduced using a plasmid transfection approach in cell culture. When miR-223 was inhibited, there was increase in viral titre in supernatants and lysates of infected EAhy926 cells. Employing miRNA target prediction software programs (miRanda, PicTar, TargetScan), the candidate stathmin 1 (STMN1), a microtubule destabilizer, was identified and validated by luciferase reporter assay. Concomitant with miR-223 down-regulation, there was a 63-fold increase in the level of STMN1 mRNA in infected cells. Likewise, the overexpression of STMN1 increased viral replication in culture supernatants and lysates. In conclusion, dengue infection was able to decrease miR-223 levels, which in turn increases STMN1 protein expression and improves virus replication, suggesting a potential antiviral role for this miRNA [127].

To identify miRNAs related to dengue pathophysiological mechanisms, the miRNA profile from blood samples of dengue-infected patients was compared with that of healthy individuals and patients with influenza. As a result, 12 miRNAs (miR-1204, miR-1225-5p, miR-3121-3p, miR-4259, miR-4327, miR-450b-5p, miR-491-5p, miR-499a-3p, miR-512-5p, miR-615-5p, miR-624-5p, miR-892b) showing an expression profile specific to acute dengue and fourteen miRNAs (miR-99b-5p, miR-218-5p, miR-296-5p, miR-499a-5p, miR-520d-3p, miR-520 g, miR-524-3p, miR-544a, miR-548 h-5p, miR-625-3p, miR-649, miR-1181, miR-1277-3p, miR-3937) that were similarly altered in dengue and influenza were identified. Additionally, 17 miRNAs presenting consistent differential expression between different clinical presentations (dengue fever, dengue fever with liver complications or dengue fever with haemorrhagic fever), which can be observed during dengue infection, were also identified. For more detailed information, see [128]. In the pathway analysis of the panel of 12 miRNAs showing acute dengue-specific expression, the most affected pathways were the PI3K-AKT survival pathway, JAK-STAT and cytokine-cytokine receptor signalling. This result is consistent with available data on some of the targets of altered miRNAs, such as miR-1204, which is related to cell death induction, miR-491-5p, which is a negative regulator of p53 and Bcl-XL, and miR-512-5p, which represses nucleotide-binding oligomerization domain containing 2 (NOD2). No functional characterization experiments were carried out [128].

Taken together, the research studies cited above reveal that the different human miRNAs produced during DENV infection may have proviral [118] and antiviral [123, 124] roles or are associated with the severity of disease through regulation of cytokine expression [125, 126].

### Japanese encephalitis virus

JEV infection targets the central nervous system and induces a profound neuroinflammation. To evaluate the role of host miRNAs in JEV infection, Zhu et al. [129] performed a deep-sequencing analysis of JEV-infected astrocytes (U251). Among the miRNAs observed to be up-regulated during infection, miR-15b was chosen for further investigation because it had been previously linked to inflammation pathways. miR-15b expression was shown to be induced after JEV infection (U251 and BV-2 cells) in a time- and MOI-dependent fashion. In addition, miR-15b mimics induced a higher expression of several inflammatory cytokines (TNF-α, IL-1β, IL-6, CCL5, IL-12p70) and ISGs (IFN-β, ISG56, OAS1, MxA and ISG15) while cells treated with a miR-15b inhibitor exhibited the opposite results. Additionally, with mir-15b mimics or inhibitors, no effect on viral replication was noted. In search of cellular targets of miR-15b, a negative regulator of retinoic acid-inducible gene 1 (RIG-I), ring finger protein 125 (RNF125), was identified by computational prediction. This finding was experimentally confirmed as well as the modulation of RIG-I expression during JEV infection. The results suggested an inverse correlation of miR-15-b and RIG-I expression with RNF125. The effect on the inflammatory response was also evaluated by measuring cytokine production after inducing or repressing the expression of miR-15b and/or RNF125 followed by JEV infection. The results show a direct link among miR-15b up-regulation during JEV infection, RNF125 suppression and inflammatory cytokine response. To validate the in vitro results, the miR-15b study was extended to a mouse model of JEV infection. In infected mice, high miR-15b and low RNF125 levels were found. In the presence of a miR-15b antagomir, the concentration of IL-1β, IL-6 and CCL2 in brain samples was significantly decreased. In addition, the antagomir treatment improved mouse survival, controlled viral replication and prevented brain damage. In summary, miR-15b is a strong candidate for therapeutic intervention [129].

Using a miRNA PCR array covering 84 miRNA sequences, Thounaojam et al. [130] investigated the JEV-induced (GP78 strain) mouse microglial (BV-2 cell line) miRNA profile. The assay results revealed four highly up-regulated miRNAs (up to 60-fold change) at 24 hpi: miR-29b, miR-155, miR-301a and miR-301b. Because miR-29b showed the highest modulation and has already been assigned to a pro-inflammatory role, it was selected for further investigations. miR-29b up-regulation was confirmed in JEV-infected primary microglial cells (from BALB/c mice). Furthermore, tumour necrosis factor alpha-induced protein 3 (TNFAIP3), a negative regulator of NF-kB and the presumed target of mir-29b, was shown to be down-regulated at the mRNA and protein levels. Treatment of infected cells with a miR-29b mimic or an anti-miR-29b confirmed the targeting of TNFAIP3. The miR-29b production during in vitro JEV infection was shown to be associated with an increase in the expression of microglial activation markers, such as inducible nitric oxide synthase and cyclooxygenase-2, pro-inflammatory cytokine production and NF-kB pathway activation. Thus, it can be inferred that miR-29b contributes to production of pro-inflammatory mediators and the activation of microglial cells, a key event in JEV-induced neuroinflammation [130]. Next, Thounaojam et al. [131] evaluated the role of host miR-155 in the regulation of the inflammatory response during JEV (GP78 strain) infection. miR-155 overexpression was observed in the brain of JEV-infected mice (3, 5 and 7 dpi - BALB/c), in post-mortem human brain samples of JEV confirmed cases and in JEV-infected BV-2 and primary microglial cells (24 hpi). Employing bioinformatics tools (Pictar and TargetScan), the Src homology 2-containing inositol phosphatase 1 (SHIP1) mRNA was predicted to be targeted by miR-155. In JEV-infected BV-2 cells and in the brains of infected mice, a time-dependent decrease in the expression levels of SHIP1 mRNA was observed. Similarly, addition of a miR-155 inhibitor to JEV-infected BV-2 cells and mice led to an increase in SHIP1 expression. Thus, JEV infection resulted in the up-regulation of miR-155 and down-regulation of SHIP1 mRNA expression. JEV infection promotes the secretion of pro-inflammatory cytokines, which is related to microglia activation and neuronal death in the mouse brain. Infected mice that were treated with an inhibitor of miR-155 showed a decrease in the production of IFN-β, TNF-α, MCP-1 and IL-6, an increase in SHIP1 expression, a decrease in symptoms associated to JEV infection and an increase in mouse survival. In conclusion, the authors suggest miR-155 as a potential therapeutic target in JEV infection, as it promotes inflammation by inhibiting SHIP1, a negative regulator of IFN-β [131].

Pareek et al. [132], on the other hand, identified miR-155 as an inhibitor of JEV replication and activation of microglia cells. When human microglial cells (CHME3) infected with JEV (P20778 strain) were evaluated with a global miRNA array to identify differentially expressed miRNAs, miR-155 was shown to be down-regulated early in infection (6 hpi) but was up-regulated later on (24 and 48 hpi). When miR-155 was overexpressed in CHME3 cells, through miR-155 mimic transfection, followed by JEV infection, NS1 protein, JEV RNA and JEV titre were significantly reduced compared to controls (mimic control transfection followed by JEV infection). In addition, human microglial cells overexpressing miR-155 that were infected with JEV showed a reduction in IFN-β expression, ISG transcripts, IRF8 expression and NF-kB activation compared to JEV infection alone [132]. The contradictory results obtained by Pareek et al. [132] and Thounaojam et al. [131] may be attributed to differences in the cells studied (CHME3 versus BV-2 and mouse brain) or in the JEV strains employed in the different studies (P20778 versus slow-growing GP78) and/or to the amount of miR-155 produced during the infections [132].

Pareek et al. [132] also studied miR-146a, which in an initial analysis had been shown to be up-regulated at an early stage (6 hpi) and down-regulated at later time points (24 and 48 hpi) post infection; however, no significant effect was attributed to it in this study. Sharma et al. [133], on the other hand, described high levels of miR-146a after the infection of CHME3 cells with JEV JaOArS982 strain (24 hpi). To verify if this was a strain-specific effect, miR-146a production in response to the infection of CHME3 cells with two different JEV strains, P20778 Vellore and JaOArS982, was compared. The results from Sharma et al. [133] regarding miR-146a expression after JEV P20778 strain infection were consistent with those previously obtained by Pareek et al. [132], confirming this was a strain-specific effect. Further studies on the effects of the miR-146a increase in JEV-infected CHME3 cells showed that this miRNA overexpression was associated with increased amounts of JEV RNA, especially at 24 hpi (JaOArS982). In addition, when cell cultures were treated with anti-miR-146a, prior to JEV infection (JaOArS982), the viral copy number decreased, suggesting a positive effect of miR-146a on JEV replication. TRAF6, IRAK1, and IRAK2, which are involved in NF-kB pathway activation, are reported to be miR-146a targets. Both JEV infection (JaOArS982) and miR-146a overexpression resulted in the down-regulation of these genes. Silencing of miR-146a prior to JEV infection (JaOArS982), on the other hand, was able to rescue their expression. In addition, miRNA-146a targeted the signal transducer and activator of transcription 1 (STAT1), which is important in promoting interferon-stimulated response elements and ISG expression. Summing up, in this model of JEV infection (JaOArS982 strain), miR-146a up-regulation was shown to be associated with inhibition of the NF-kB pathway activation and disruption of antiviral pathway signalling [133].

Finally, Cai et al. [134] investigated the host miRNA production in JEV-infected porcine kidney epithelial cells (PK15). Among the 132 differentially expressed (≥ 2-fold change) miRNAs, 59 % were up-regulated in infected compared to uninfected cells, and 41 % were down-regulated. Many of these had already been reported to be modulated in response to other viral infections. Target prediction of the differentially expressed miRNAs and gene ontology analysis of the predicted targets was carried out, but no functional assays were performed [134].

### Closing remarks on host miRNAs modulated during flavivirus infection

It is likely that most of the human and mouse flavivirus-induced miRNAs are produced in a virus-specific and host-dependent fashion. Currently, the only human miRNA induced by different flaviviruses, including JEV and DENV, is miR-146a, which was also found in other virus infection models, such as vesicular stomatitis virus [135], Hendra virus [136], enterovirus [117], hepatitis B virus [137, 138] and human neurotrophic viruses [139], among others. Mammalian cells infected with enterovirus 71 [117], dengue virus 2 [118], JEV [133] or vesicular stomatitis virus [135] produced miRNA-146a. This miRNA facilitates viral replication by targeting TRAF6, which has an essential role in the signalling pathways of Toll-like or RIG-I like receptors [117, 118, 133, 135]. Although common miRNAs have been identified as differentially expressed in the studies of Kumar and Nerurkar [122] and Cai et al. [134] in WNV and JEV infection models, respectively, none of these were confirmed through functional assays, such as the use of miRNA mimics or inhibitors.

In sum, identifying virus-induced human miRNAs may be a good approach to search for biomarkers that could be used for disease diagnosis, prevention and treatment [140].

## Conclusion

In conclusion, regarding the *Flavivirus* genus, by the time this review was finished, there was experimental evidence for the existence of viral miRNAs derived from WNV and DENV and viral sfRNA from all members of this genus where it has been investigated.

The identification of viral miRNAs with immunomodulatory activities increases the potential targets for therapeutic approaches. On the other hand, molecules that “neutralize” virus-derived miRNAs could be used to eradicate miRNA-mediated escape, favouring the course of the immune response to the viral infection. Additionally, finding viral mutants in miRNA sequences or viruses that are defective in the production of sfRNAs may provide information for the rational development of vaccines using attenuated flaviviruses. Moreover, the discovery and characterization of viral miRNAs/sfRNAs contribute to the development of tools for studying the cellular mechanisms involved in virus-host interaction, viral RNA turnover and viral pathogenicity associated with flavivirus infection.

Both types of molecules, miRNAs and sfRNAs, are strongly associated with the modulation of host cell gene expression and immune response during flavivirus infection. Human miRNAs are also closely linked to the regulation of viral replication. Taking this into account, both human and viral miRNAs could play an important role as biomarkers of viral infection prognosis and pathogenicity, as well as diagnostic tools and potential therapeutic targets.

## Abbreviations

Ago, argonaute; AKT, RAC -alpha serine/threonine-protein kinase; ARMC8, armadillo repeat containing 8; BACH1, BTB and CNC homology 1, basic leucine zipper transcription factor 1; bp, base pair; C, capsid; CAPRIN1, cell cycle associated protein 1; CCL, C-C motif chemokine ligand; CPEB3, cytoplasmic polyadenylation element binding protein 3; CTCF, CCCTC binding factor; DENV, dengue virus; DF, dengue fever; DGCR8, DiGeorge critical region 8; DHF, dengue haemorrhagic fever; dpi, days post-infection; dsRNA, double stranded RNA; DSS, dengue shock syndrome; E, envelope; ECOP, EGFR-coamplified and overexpressed protein; EZH2, enhancer of zeste 2 polycomb repressive complex 2 subunit; FAM116A, family with sequence similarity 116, member A; G3BP, GTPase Activating Protein (SH3 Domain) Binding Protein; GATA4, transcription factor GATA-4; GBP1, guanylate binding protein 1; gRNA, genomic RNA; HO-1, haeme oxygenase-1; hpi, hours post-infection; IFN, interferon; IL, interleukin; IRAK, interleukin 1 receptor associated kinase; IRF, interferon regulatory factor; ISG, interferon-stimulated gene; ISG15, interferon-stimulated protein, 15 kDa; ISG56, interferon-induced 56 kDa protein; IkBα, nuclear factor of kappa light polypeptide gene enhancer in B-cells inhibitor, alpha; JAK, Janus kinase; JEV, Japanese encephalitis virus; Ldbr, lariat-debranching enzyme; M, membrane; MAVS, mitochondrial antiviral signalling protein; MCP-1, Monocyte chemoattractant protein-1; MEF, Mouse embryonic fibroblast; mESCs, mouse embryonic stem cells; MFE, minimum free energy; miR genes, miRNA genes; miRNA, microRNA; MOI, multiplicity of infection; MVEV, Murray Valley encephalitis virus; MxA, myxoma resistance protein 1; ncRNA, non-coding RNA; NF-kB, nuclear factor kappa B; NOD2, nucleotide-binding oligomerization domain containing 2; NS, nonstructural; nt, nucleotide; OAS1, 2′-5′-oligoadenylate synthetase 1; ORF, open reading frame; PACT, protein activator of the interferon-induced protein kinase; PBMC, peripheral blood mononuclear *cells*; *PBS*, *primer binding sequence*; PI3K, phosphatidyl inositol 3-kinase; piRNA, PIWI pathway small RNA; pre-miRNA, precursor miRNA; pre-RISC, pre-RNA induced silencing complex; pre-tRNA, transfer RNA precursor; pri-miRNA, primary miRNA; prM, pre-membrane; qPCR, quantitative polymerase chain reaction; qRT-PCR, quantitative reverse transcription polymerase chain reaction; RAB11A, RAB11A, member RAS oncogene family; RASSF5, Ras association domain family member 5; RBP, RNA-binding protein; RIG-I, retinoic acid-inducible gene 1; RNAi, RNA interference; RNF125, ring finger protein 125; RT-PCR, reverse transcription polymerase chain reaction; SD junction, ssRNA-dsRNA junction; SESTD1, SEC14 and spectrin domains 1; sfRNA, subgenomic flavivirus RNA; SHIP1, Src homology 2-containing inositol phosphatase 1; shRNA, short hairpin RNA; siRNA, small interfering RNA; SLEV, Saint Louis encephalitis virus; SMAD2, SMAD family member 2; snoRNA, small nucleolar RNA; SOCS1, suppressor of cytokine signalling 1; sRNA, small RNA; SRV, Saumarez Reef virus; ssRNA, single stranded RNA; STAT, signal transducer and activator of transcription; STMN1, stathmin 1; TAB3, TGF-beta activated kinase 1/MAP3K7 binding protein 3; TBEV, tick-borne encephalitis virus; TLR, toll-like receptor; TMD3, transmembrane domain 3; TNFAIP3, tumour necrosis factor alpha-induced protein 3; TNF-α, tumor necrosis factor alpha; TRAF6, TNF receptor-associated factor 6; TRBP, human immunodeficiency virus (HIV)-1 transactivating response (TAR) RNA-binding protein; TRIM25, tripartite motif-containing 25; usRNA, unusually small RNA; UTR, untranslated region; vsRNA, virus-derived small RNA; WNV, West Nile virus; XRN1, 5′- 3′ exoribonuclease 1; YFV, yellow fever virus; ZNF536, zinc finger protein 536.

## References

[1] Lee RC, Feinbaum RL, Ambros V (1993). The C. elegans heterochronic gene lin-4 encodes small RNAs with antisense complementarity to lin-14. Cell.

[2] Reinhart BJ, Slack FJ, Basson M, Pasquinelli AE, Bettinger JC, Rougvie AE (2000). The 21-nucleotide let-7 RNA regulates developmental timing in Caenorhabditis elegans. Nature.

[3] Pasquinelli AE, Reinhart BJ, Slack F, Martindale MQ, Kuroda MI, Maller B (2000). Conservation of the sequence and temporal expression of let-7 heterochronic regulatory RNA. Nature.

[4] Dogini DB, Pascoal VD, Avansini SH, Vieira AS, Pereira TC, Lopes-Cendes I (2014). The new world of RNAs. Genet Mol Biol.

[5] Browse miRBase by species. http://www.mirbase.org/cgi-bin/browse.pl?org=hsa. Accessed 11 May 2016.

[6] Friedlander MR, Lizano E, Houben AJ, Bezdan D, Banez-Coronel M, Kudla G (2014). Evidence for the biogenesis of more than 1,000 novel human microRNAs. Genome Biol.

[7] Kim VN, Han J, Siomi MC (2009). Biogenesis of small RNAs in animals. Nat Rev Mol Cell Biol.

[8] Ozsolak F, Poling LL, Wang Z, Liu H, Liu XS, Roeder RG (2008). Chromatin structure analyses identify miRNA promoters. Genes Dev.

[9] Monteys AM, Spengler RM, Wan J, Tecedor L, Lennox KA, Xing Y (2010). Structure and activity of putative intronic miRNA promoters. RNA.

[10] Tal TL, Tanguay RL (2012). Non-coding RNAs--novel targets in neurotoxicity. Neurotoxicology.

[11] miRBase: the microRNA database. http://www.mirbase.org/. Accessed 11 May 2016.

[12] Grundhoff A, Sullivan CS (2011). Virus-encoded microRNAs. Virology.

[13] Pfeffer S, Zavolan M, Grässer FA, Chien M, Russo JJ, Ju J (2004). Identification of virus-encoded microRNAs. Science.

[14] Klase Z, Kale P, Winograd R, Gupta MV, Heydarian M, Berro R (2007). HIV-1 TAR element is processed by Dicer to yield a viral micro-RNA involved in chromatin remodeling of the viral LTR. BMC Mol Biol.

[15] Hussain M, Torres S, Schnettler E, Funk A, Grundhoff A, Pijlman GP (2012). West Nile virus encodes a microRNA-like small RNA in the 3′ untranslated region which up-regulates GATA4 mRNA and facilitates virus replication in mosquito cells. Nucleic Acids Res.

[16] Hussain M, Asgari S (2014). MicroRNA-like viral small RNA from Dengue virus 2 autoregulates its replication in mosquito cells. Proc Natl Acad Sci U S A.

[17] Lee Y, Kim M, Han J, Yeom KH, Lee S, Baek SH (2004). MicroRNA genes are transcribed by RNA polymerase II. EMBO J.

[18] Lee Y, Jeon K, Lee JT, Kim S, Kim VN (2002). MicroRNA maturation: stepwise processing and subcellular localization. EMBO J.

[19] Gregory RI, Yan KP, Amuthan G, Chendrimada T, Doratotaj B, Cooch N (2004). The Microprocessor complex mediates the genesis of microRNAs. Nature.

[20] Kim YK, Kim VN (2007). Processing of intronic microRNAs. EMBO J.

[21] Pawlicki JM, Steitz JA (2008). Primary microRNA transcript retention at sites of transcription leads to enhanced microRNA production. J Cell Biol.

[22] Han J, Lee Y, Yeom KH, Nam JW, Heo I, Rhee JK (2006). Molecular basis for the recognition of primary microRNAs by the Drosha-DGCR8 complex. Cell.

[23] Lee Y, Ahn C, Han J, Choi H, Kim J, Yim J (2003). The nuclear RNase III Drosha initiates microRNA processing. Nature.

[24] Denli AM, Tops BB, Plasterk RH, Ketting RF, Hannon GJ (2004). Processing of primary microRNAs by the Microprocessor complex. Nature.

[25] Yi R, Qin Y, Macara IG, Cullen BR (2003). Exportin-5 mediates the nuclear export of pre-microRNAs and short hairpin RNAs. Genes Dev.

[26] Kawamata T, Tomari Y (2010). Making RISC. Trends Biochem Sci.

[27] Liu J, Carmell MA, Rivas FV, Marsden CG, Thomson JM, Song JJ (2004). Argonaute2 is the catalytic engine of mammalian RNAi. Science.

[28] Bartel DP (2009). MicroRNAs: target recognition and regulatory functions. Cell.

[29] Lewis BP, Shih IH, Jones-Rhoades MW, Bartel DP, Burge CB (2003). Prediction of mammalian microRNA targets. Cell.

[30] Rigoutsos I (2009). New tricks for animal microRNAS: targeting of amino acid coding regions at conserved and nonconserved sites. Cancer Res.

[31] Abdelfattah AM, Park C, Choi MY (2014). Update on non-canonical microRNAs. Biomol Concepts.

[32] Ha M, Kim VN (2014). Regulation of microRNA biogenesis. Nat Rev Mol Cell Biol.

[33] Bartel DP (2004). MicroRNAs: genomics, biogenesis, mechanism, and function. Cell.

[34] Hammond SM (2005). Dicing and slicing: the core machinery of the RNA interference pathway. FEBS Lett.

[35] Lee YS, Nakahara K, Pham JW, Kim K, He Z, Sontheimer EJ (2004). Distinct roles for Drosophila Dicer-1 and Dicer-2 in the siRNA/miRNA silencing pathways. Cell.

[36] Brackney DE, Scott JC, Sagawa F, Woodward JE, Miller NA, Schilkey FD (2010). C6/36 Aedes albopictus cells have a dysfunctional antiviral RNA interference response. PLoS Negl Trop Dis.

[37] Vidigal JA, Ventura A (2015). The biological functions of miRNAs: lessons from in vivo studies. Trends Cell Biol.

[38] Eiring AM, Harb JG, Neviani P, Garton C, Oaks JJ, Spizzo R (2010). miR-328 functions as an RNA decoy to modulate hnRNP E2 regulation of mRNA translation in leukemic blasts. Cell.

[39] Li M, Marin-Muller C, Bharadwaj U, Chow KH, Yao Q, Chen C (2009). MicroRNAs: control and loss of control in human physiology and disease. World J Surg.

[40] Mu P, Han YC, Betel D, Yao E, Squatrito M, Ogrodowski P (2009). Genetic dissection of the miR-17 ~ 92 cluster of microRNAs in Myc-induced B-cell lymphomas. Genes Dev.

[41] Olive V, Bennett MJ, Walker JC, Ma C, Jiang I, Cordon-Cardo C (2009). miR-19 is a key oncogenic component of mir-17-92. Genes Dev.

[42] Abd-El-Fattah AA, Sadik NA, Shaker OG, Aboulftouh ML (2013). Differential microRNAs expression in serum of patients with lung cancer, pulmonary tuberculosis, and pneumonia. Cell Biochem Biophys.

[43] Akamatsu S, Hayes CN, Tsuge M, Miki D, Akiyama R, Abe H (2015). Differences in serum microRNA profiles in hepatitis B and C virus infection. J Infect.

[44] Heneghan HM, Miller N, Kelly R, Newell J, Kerin MJ (2010). Systemic miRNA-195 differentiates breast cancer from other malignancies and is a potential biomarker for detecting noninvasive and early stage disease. Oncologist.

[45] Anokye-Danso F, Snitow M, Morrisey EE (2012). How microRNAs facilitate reprogramming to pluripotency. J Cell Sci.

[46] Card DA, Hebbar PB, Li L, Trotter KW, Komatsu Y, Mishina Y (2008). Oct4/Sox2-regulated miR-302 targets cyclin D1 in human embryonic stem cells. Mol Cell Biol.

[47] Cheloufi S, Dos Santos CO, Chong MM, Hannon GJ (2010). A dicer-independent miRNA biogenesis pathway that requires Ago catalysis. Nature.

[48] Zhang X, Wang H, Zhang S, Song J, Zhang Y, Wei X (2012). MiR-134 functions as a regulator of cell proliferation, apoptosis, and migration involving lung septation. In Vitro Cell Dev Biol Anim.

[49] Guo L, Xu J, Qi J, Zhang L, Wang J, Liang J (2013). MicroRNA-17-92a upregulation by estrogen leads to Bim targeting and inhibition of osteoblast apoptosis. J Cell Sci.

[50] Mondanizadeh M, Arefian E, Mosayebi G, Saidijam M, Khansarinejad B, Hashemi SM (2015). MicroRNA-124 Regulates Neuronal Differentiation of Mesenchymal Stem Cells by Targeting Sp1 mRNA. J Cell Biochem.

[51] Esguerra JL, Mollet IG, Salunkhe VA, Wendt A, Eliasson L (2014). Regulation of pancreatic beta cell stimulus-secretion coupling by microRNAs. Genes (Basel).

[52] Vickers KC, Landstreet SR, Levin MG, Shoucri BM, Toth CL, Taylor RC (2014). MicroRNA-223 coordinates cholesterol homeostasis. Proc Natl Acad Sci U S A.

[53] Allantaz F, Cheng DT, Bergauer T, Ravindran P, Rossier MF, Ebeling M (2012). Expression profiling of human immune cell subsets identifies miRNA-mRNA regulatory relationships correlated with cell type specific expression. PLoS One.

[54] Nukui M, Mori Y, Murphy EA (2015). A human herpesvirus 6A-encoded microRNA: role in viral lytic replication. J Virol.

[55] Fabbri M, Paone A, Calore F, Galli R, Gaudio E, Santhanam R (2012). MicroRNAs bind to Toll-like receptors to induce prometastatic inflammatory response. Proc Natl Acad Sci U S A.

[56] Boss IW, Renne R (2011). Viral miRNAs and immune evasion. Biochim Biophys Acta.

[57] Fa M, Schlesinger R (1980). Togavirus morphology and morphogenesis. The togaviruses: biology, structure, replication.

[58] Chambers TJ, Hahn CS, Galler R, Rice CM (1990). Flavivirus genome organization, expression, and replication. Annu Rev Microbiol.

[59] Brinton MA, Basu M (2015). Functions of the 3′ and 5′ genome RNA regions of members of the genus Flavivirus. Virus Res.

[60] Cahour A, Pletnev A, Vazielle-Falcoz M, Rosen L, Lai CJ (1995). Growth-restricted dengue virus mutants containing deletions in the 5′ noncoding region of the RNA genome. Virology.

[61] Proutski V, Gritsun TS, Gould EA, Holmes EC (1999). Biological consequences of deletions within the 3′-untranslated region of flaviviruses may be due to rearrangements of RNA secondary structure. Virus Res.

[62] Cui T, Sugrue RJ, Xu Q, Lee AK, Chan YC, Fu J (1998). Recombinant dengue virus type 1 NS3 protein exhibits specific viral RNA binding and NTPase activity regulated by the NS5 protein. Virology.

[63] Shi PY, Li W, Brinton MA (1996). Cell proteins bind specifically to West Nile virus minus-strand 3′ stem-loop RNA. J Virol.

[64] Hahn CS, Hahn YS, Rice CM, Lee E, Dalgarno L, Strauss EG (1987). Conserved elements in the 3′ untranslated region of flavivirus RNAs and potential cyclization sequences. J Mol Biol.

[65] Proutski V, Gould EA, Holmes EC (1997). Secondary structure of the 3′ untranslated region of flaviviruses: similarities and differences. Nucleic Acids Res.

[66] Olsthoorn RC, Bol JF (2001). Sequence comparison and secondary structure analysis of the 3′ noncoding region of flavivirus genomes reveals multiple pseudoknots. RNA.

[67] Gritsun TS, Gould EA (2006). Direct repeats in the 3′ untranslated regions of mosquito-borne flaviviruses: possible implications for virus transmission. J Gen Virol.

[68] Rice CM, Lenches EM, Eddy SR, Shin SJ, Sheets RL, Strauss JH (1985). Nucleotide sequence of yellow fever virus: implications for flavivirus gene expression and evolution. Science.

[69] Rouha H, Thurner C, Mandl CW (2010). Functional microRNA generated from a cytoplasmic RNA virus. Nucleic Acids Res.

[70] Shapiro JS, Langlois RA, Pham AM, Tenoever BR (2012). Evidence for a cytoplasmic microprocessor of pri-miRNAs. RNA.

[71] Gaglia MM, Glaunsinger BA (2010). Viruses and the cellular RNA decay machinery. Wiley Interdiscip Rev RNA.

[72] Moon SL, Barnhart MD, Wilusz J (2012). Inhibition and avoidance of mRNA degradation by RNA viruses. Curr Opin Microbiol.

[73] Moon SL, Wilusz J (2013). Cytoplasmic viruses: rage against the (cellular RNA decay) machine. PLoS Pathog.

[74] Green AM, Beatty PR, Hadjilaou A, Harris E (2014). Innate immunity to dengue virus infection and subversion of antiviral responses. J Mol Biol.

[75] Roby JA, Pijlman GP, Wilusz J, Khromykh AA (2014). Noncoding subgenomic flavivirus RNA: multiple functions in West Nile virus pathogenesis and modulation of host responses. Viruses.

[76] Moon SL, Anderson JR, Kumagai Y, Wilusz CJ, Akira S, Khromykh AA (2012). A noncoding RNA produced by arthropod-borne flaviviruses inhibits the cellular exoribonuclease XRN1 and alters host mRNA stability. RNA.

[77] Charley PA, Wilusz J (2015). Standing your ground to exoribonucleases: Function of Flavivirus long non-coding RNAs. Virus Res.

[78] Moon SL, Dodd BJ, Brackney DE, Wilusz CJ, Ebel GD, Wilusz J (2015). Flavivirus sfRNA suppresses antiviral RNA interference in cultured cells and mosquitoes and directly interacts with the RNAi machinery. Virology.

[79] Urosevic N, van Maanen M, Mansfield JP, Mackenzie JS, Shellam GR (1997). Molecular characterization of virus-specific RNA produced in the brains of flavivirus-susceptible and -resistant mice after challenge with Murray Valley encephalitis virus. J Gen Virol.

[80] Lin KC, Chang HL, Chang RY (2004). Accumulation of a 3′-terminal genome fragment in Japanese encephalitis virus-infected mammalian and mosquito cells. J Virol.

[81] Scherbik SV, Paranjape JM, Stockman BM, Silverman RH, Brinton MA (2006). RNase L plays a role in the antiviral response to West Nile virus. J Virol.

[82] Pijlman GP, Funk A, Kondratieva N, Leung J, Torres S, van der Aa L (2008). A highly structured, nuclease-resistant, noncoding RNA produced by flaviviruses is required for pathogenicity. Cell Host Microbe.

[83] Funk A, Truong K, Nagasaki T, Torres S, Floden N, Balmori Melian E (2010). RNA structures required for production of subgenomic flavivirus RNA. J Virol.

[84] Chapman EG, Moon SL, Wilusz J, Kieft JS (2014). RNA structures that resist degradation by Xrn1 produce a pathogenic Dengue virus RNA. Elife.

[85] Chapman EG, Costantino DA, Rabe JL, Moon SL, Wilusz J, Nix JC (2014). The structural basis of pathogenic subgenomic flavivirus RNA (sfRNA) production. Science.

[86] Colpitts TM, Conway MJ, Montgomery RR, Fikrig E (2012). West Nile Virus: biology, transmission, and human infection. Clin Microbiol Rev.

[87] Schuessler A, Funk A, Lazear HM, Cooper DA, Torres S, Daffis S (2012). West Nile virus noncoding subgenomic RNA contributes to viral evasion of the type I interferon-mediated antiviral response. J Virol.

[88] Schnettler E, Sterken MG, Leung JY, Metz SW, Geertsema C, Goldbach RW (2012). Noncoding flavivirus RNA displays RNA interference suppressor activity in insect and Mammalian cells. J Virol.

[89] Parameswaran P, Sklan E, Wilkins C, Burgon T, Samuel MA, Lu R (2010). Six RNA viruses and forty-one hosts: viral small RNAs and modulation of small RNA repertoires in vertebrate and invertebrate systems. PLoS Pathog.

[90] Guzman MG, Halstead SB, Artsob H, Buchy P, Farrar J, Gubler DJ (2010). Dengue: a continuing global threat. Nat Rev Microbiol.

[91] Liu R, Yue L, Li X, Yu X, Zhao H, Jiang Z (2010). Identification and characterization of small sub-genomic RNAs in dengue 1-4 virus-infected cell cultures and tissues. Biochem Biophys Res Commun.

[92] Liu Y, Liu H, Zou J, Zhang B, Yuan Z (2014). Dengue virus subgenomic RNA induces apoptosis through the Bcl-2-mediated PI3k/Akt signaling pathway. Virology.

[93] Bidet K, Dadlani D, Garcia-Blanco MA (2014). G3BP1, G3BP2 and CAPRIN1 are required for translation of interferon stimulated mRNAs and are targeted by a dengue virus non-coding RNA. PLoS Pathog.

[94] Bidet K, Garcia-Blanco MA (2014). Flaviviral RNAs: weapons and targets in the war between virus and host. Biochem J.

[95] Manokaran G, Finol E, Wang C, Gunaratne J, Bahl J, Ong EZ (2015). Dengue subgenomic RNA binds TRIM25 to inhibit interferon expression for epidemiological fitness. Science.

[96] Kakumani PK, Ponia SS, RK S, Sood V, Chinnappan M, Banerjea AC (2013). Role of RNA interference (RNAi) in dengue virus replication and identification of NS4B as an RNAi suppressor. J Virol.

[97] Scott JC, Brackney DE, Campbell CL, Bondu-Hawkins V, Hjelle B, Ebel GD (2010). Comparison of dengue virus type 2-specific small RNAs from RNA interference-competent and -incompetent mosquito cells. PLoS Negl Trop Dis.

[98] Hess AM, Prasad AN, Ptitsyn A, Ebel GD, Olson KE, Barbacioru C (2011). Small RNA profiling of dengue virus-mosquito interactions implicates the PIWI RNA pathway in anti-viral defense. BMC Microbiol.

[99] Schirtzinger EE, Andrade CC, Devitt N, Ramaraj T, Jacobi JL, Schilkey F (2015). Repertoire of virus-derived small RNAs produced by mosquito and mammalian cells in response to dengue virus infection. Virology.

[100] Bogerd HP, Skalsky RL, Kennedy EM, Furuse Y, Whisnant AW, Flores O (2014). Replication of many human viruses is refractory to inhibition by endogenous cellular microRNAs. J Virol.

[101] Ospina-Bedoya M, Campillo-Pedroza N, Franco-Salazar JP, Gallego-Gomez JC (2014). Computational Identification of dengue virus microRNA-Like Structures and their Cellular Targets. Bioinform Biol Insights.

[102] van den Hurk AF, Ritchie SA, Mackenzie JS (2009). Ecology and geographical expansion of Japanese encephalitis virus. Annu Rev Entomol.

[103] Fan YH, Nadar M, Chen CC, Weng CC, Lin YT, Chang RY (2011). Small noncoding RNA modulates Japanese encephalitis virus replication and translation in trans. Virol J.

[104] Chang RY, Hsu TW, Chen YL, Liu SF, Tsai YJ, Lin YT (2013). Japanese encephalitis virus non-coding RNA inhibits activation of interferon by blocking nuclear translocation of interferon regulatory factor 3. Vet Microbiol.

[105] Knox J, Cowan RU, Doyle JS, Ligtermoet MK, Archer JS, Burrow JN (2012). Murray Valley encephalitis: a review of clinical features, diagnosis and treatment. Med J Aust.

[106] Selvey LA, Dailey L, Lindsay M, Armstrong P, Tobin S, Koehler AP (2014). The changing epidemiology of Murray Valley encephalitis in Australia: the 2011 outbreak and a review of the literature. PLoS Negl Trop Dis.

[107] Tomori O (2004). Yellow fever: the recurring plague. Crit Rev Clin Lab Sci.

[108] Barnett ED (2007). Yellow fever: epidemiology and prevention. Clin Infect Dis.

[109] Silva PA, Pereira CF, Dalebout TJ, Spaan WJ, Bredenbeek PJ (2010). An RNA pseudoknot is required for production of yellow fever virus subgenomic RNA by the host nuclease XRN1. J Virol.

[110] Silva PAGC (2011). Functions and requirements of conserved RNA structures in the 3′ untranslated region of Flaviviruses.

[111] Villordo SM, Carballeda JM, Filomatori CV, Gamarnik AV (2016). RNA structure duplications and flavivirus host adaptation. Trends Microbiol.

[112] Schnettler E, Tykalová H, Watson M, Sharma M, Sterken MG, Obbard DJ (2014). Induction and suppression of tick cell antiviral RNAi responses by tick-borne flaviviruses. Nucleic Acids Res.

[113] Li SC, Shiau CK, Lin WC (2008). Vir-Mir db: prediction of viral microRNA candidate hairpins. Nucleic Acids Res.

[114] Vir-Mir database. http://alk.ibms.sinica.edu.tw/cgi-bin/miRNA/virus_tax.cgi?tax_id=35278. Accessed 29 Mar 2015.

[115] Brackney DE, Beane JE, Ebel GD (2009). RNAi targeting of West Nile virus in mosquito midguts promotes virus diversification. PLoS Pathog.

[116] Bryant JE, Vasconcelos PF, Rijnbrand RC, Mutebi JP, Higgs S, Barrett AD (2005). Size heterogeneity in the 3′ noncoding region of South American isolates of yellow fever virus. J Virol.

[117] Ho BC, Yu IS, Lu LF, Rudensky A, Chen HY, Tsai CW (2014). Inhibition of miR-146a prevents enterovirus-induced death by restoring the production of type I interferon. Nat Commun.

[118] Wu S, He L, Li Y, Wang T, Feng L, Jiang L (2013). miR-146a facilitates replication of dengue virus by dampening interferon induction by targeting TRAF6. J Infect.

[119] Umbach JL, Cullen BR (2009). The role of RNAi and microRNAs in animal virus replication and antiviral immunity. Genes Dev.

[120] Smith JL, Grey FE, Uhrlaub JL, Nikolich-Zugich J, Hirsch AJ (2012). Induction of the cellular microRNA, Hs_154, by West Nile virus contributes to virus-mediated apoptosis through repression of antiapoptotic factors. J Virol.

[121] Slonchak A, Shannon RP, Pali G, Khromykh AA (2015). Human miRNA miR-532-5p exhibits antiviral activity against West Nile virus via suppression of host genes SESTD1 and TAB3 required for virus replication. J Virol.

[122] Kumar M, Nerurkar VR (2014). Integrated analysis of microRNAs and their disease related targets in the brain of mice infected with West Nile virus. Virology.

[123] Zhu X, He Z, Hu Y, Wen W, Lin C, Yu J (2014). MicroRNA-30e* suppresses dengue virus replication by promoting NF-kappaB-dependent IFN production. PLoS Negl Trop Dis.

[124] Escalera-Cueto M, Medina-Martinez I, del Angel RM, Berumen-Campos J, Gutierrez-Escolano AL, Yocupicio-Monroy M (2015). Let-7c overexpression inhibits dengue virus replication in human hepatoma Huh-7 cells. Virus Res.

[125] Qi Y, Li Y, Zhang L, Huang J (2013). microRNA expression profiling and bioinformatic analysis of dengue virus-infected peripheral blood mononuclear cells. Mol Med Rep.

[126] Chen RF, Yang KD, Lee IK, Liu JW, Huang CH, Lin CY (2014). Augmented miR-150 expression associated with depressed SOCS1 expression involved in dengue haemorrhagic fever. J Infect.

[127] Wu N, Gao N, Fan D, Wei J, Zhang J, An J (2014). miR-223 inhibits dengue virus replication by negatively regulating the microtubule-destabilizing protein STMN1 in EAhy926 cells. Microbes Infect.

[128] Tambyah PA, Ching CS, Sepramaniam S, Ali JM, Armugam A, Jeyaseelan K. microRNA expression in blood of dengue patients. Ann Clin Biochem. 2015, [Epub ahead of print]. 10.1177/0004563215604001 26290515

[129] Zhu B, Ye J, Nie Y, Ashraf U, Zohaib A, Duan X (2015). MicroRNA-15b Modulates Japanese Encephalitis Virus-Mediated Inflammation via Targeting RNF125. J Immunol.

[130] Thounaojam MC, Kaushik DK, Kundu K, Basu A (2014). MicroRNA-29b modulates Japanese encephalitis virus-induced microglia activation by targeting tumor necrosis factor alpha-induced protein 3. J Neurochem.

[131] Thounaojam MC, Kundu K, Kaushik DK, Swaroop S, Mahadevan A, Shankar SK (2014). MicroRNA 155 regulates Japanese encephalitis virus-induced inflammatory response by targeting Src homology 2-containing inositol phosphatase 1. J Virol.

[132] Pareek S, Roy S, Kumari B, Jain P, Banerjee A, Vrati S (2014). MiR-155 induction in microglial cells suppresses Japanese encephalitis virus replication and negatively modulates innate immune responses. J Neuroinflammation.

[133] Sharma N, Verma R, Kumawat KL, Basu A, Singh SK (2015). miR-146a suppresses cellular immune response during Japanese encephalitis virus JaOArS982 strain infection in human microglial cells. J Neuroinflammation.

[134] Cai Y, Zhu L, Zhou Y, Liu X, Li X, Lang Q (2015). Identification and analysis of differentially-expressed microRNAs in Japanese encephalitis virus-infected PK-15 cells with deep sequencing. Int J Mol Sci.

[135] Hou J, Wang P, Lin L, Liu X, Ma F, An H (2009). MicroRNA-146a feedback inhibits RIG-I-dependent Type I IFN production in macrophages by targeting TRAF6, IRAK1, and IRAK2. J Immunol.

[136] Stewart CR, Marsh GA, Jenkins KA, Gantier MP, Tizard ML, Middleton D (2013). Promotion of Hendra virus replication by microRNA 146a. J Virol.

[137] Wang S, Zhang X, Ju Y, Zhao B, Yan X, Hu J (2013). MicroRNA-146a feedback suppresses T cell immune function by targeting Stat1 in patients with chronic hepatitis B. J Immunol.

[138] Li JF, Dai XP, Zhang W, Sun SH, Zeng Y, Zhao GY, et al. Upregulation of MicroRNA-146a by Hepatitis B Virus X Protein Contributes to Hepatitis Development by Downregulating Complement Factor H. MBio. 2015;6(2) doi: 10.1128/mBio.02459-14. 10.1128/mBio.02459-14 PMC4453536 25805734

[139] Hill JM, Clement C, Zhao Y, Lukiw WJ (2015). Induction of the pro-inflammatory NF-kB-sensitive miRNA-146a by human neurotrophic viruses. Front Microbiol.

[140] Li L, Chen XP, Li YJ (2010). MicroRNA-146a and human disease. Scand J Immunol.

